# Education reduces dementia incidence and mitigates genetic risk

**DOI:** 10.1073/pnas.2529506123

**Published:** 2026-10-23

**Authors:** Silvia H. Barcellos, Leandro S. Carvalho, Kenneth M. Langa, Sneha Nimmagadda, Patrick Turley

**Affiliations:** ^a^ La Follette School of Public Affairs, University of Wisconsin-Madison, Madison, WI 53706; ^b^ Department of Population Health Sciences, University of Wisconsin-Madison, Madison, WI 53726; ^c^ Center for Economic and Social Research, University of Southern California, Los Angeles, CA 90089; ^d^ Department of Economics, University of Southern California, Los Angeles, CA 90089; ^e^ Department of Internal Medicine, University of Michigan, Ann Arbor, MI 48109; ^f^ Survey Research Center, Survey Research Center, Institute for Social Research, University of Michigan, Ann Arbor, MI 48106

**Keywords:** education, dementia, regression discontinuity design, genetics

## Abstract

There is little causal evidence on factors that can protect individuals against Alzheimer’s Disease and Related Dementias (ADRD) risk. We study the causal effect of education on ADRD, exploiting a regression discontinuity generated by a compulsory schooling reform. ADRD was ascertained based on medical history, hospital records, and death registries, addressing concerns about selective sample attrition. We find that education reduces incidence of ADRD. Using molecular genetic data, we show that the reform weakened the relationship between genetics and ADRD incidence, implying genetic risk of dementia is not immutable and can be modified by social policy.

## Significance

Scholars have noted the need for high-quality evidence on which factors can reduce Alzheimer’s Disease and Related Dementias (ADRD) and delay its onset. Such evidence is paramount for designing policies that can prevent and effectively treat dementia. Characteristics that are highly associated with ADRD risk have been deemed as risk factors for dementia, but there is limited evidence as to whether these characteristics causally affect ADRD. We document a causal relationship between education and ADRD diagnosis.

With gains in life expectancy and population aging, cases of Alzheimer’s Disease and Related Dementias (ADRD) are sharply rising, with global cases predicted to reach 131.5 million people by 2050 (1, 2). In addition to imposing health and emotional costs on ADRD patients and their families, the severe cognitive and functional impairment that characterizes ADRD has a large economic cost. In the United States alone, ADRD’s cost is estimated at $305 billion (3). Without effective interventions to prevent or delay its onset, the economic burden of dementia could grow by as much as threefold in the next 35 y (2). For these reasons, it is crucial to understand what factors can decrease ADRD incidence and delay cognitive and functional decline in older ages.

Lower educational attainment has been identified as a major risk factor for ADRD, as education is strongly associated with better later-life cognition and lower ADRD risk (4–8). However, little is known about whether and how much of this association reflects a causal effect from education to ADRD (9, 10). Part of the challenge in distinguishing correlation from causation is that third factors, such as childhood circumstances, socioeconomic background, and genetics, may confound the relationship between education and ADRD. Also, there may be individual characteristics, such as self-control, that drive both behavioral risk factors and educational attainment.

Another obstacle to studying the education–ADRD relationship is that dementia is difficult to diagnose and measure. Cognitive decline often progresses slowly, and there is no one-to-one correspondence between brain pathology and cognition (11). Selective sample attrition is also a concern for most survey-based measures of ADRD (12) as survey participants who develop dementia are more likely to drop out.

We overcome these challenges by combining a natural experiment with dementia data constructed from administrative health records for a large cohort. In 1972, England, Scotland, and Wales increased the minimum age at which students could drop out of school from 15 to 16 y of age. The reform affected only students born after September 1, 1957, generating a discontinuity in the relationship between education and date of birth. We exploit this discontinuity using a regression discontinuity design to estimate the causal effect of education on dementia incidence.

We use data from the UK Biobank (UKB), dementia outcomes are constructed using hospital records, mortality data, and (self-reported) medical history. Crucially, these data are not biased by selective attrition; the study continues to follow its participants through linkages to administrative health and death registries.

The UKB genotyped its participants, allowing us to also investigate whether education can mitigate the genetic risk for ADRD (13). ADRD has a strong genetic basis, with an estimated twin-based heritability as high as 80% (14–16) and a SNP-heritability of 33% (17). However, it is unclear whether this genetic risk can be mitigated by behavioral changes, clinical interventions, or social policies.

Our main outcome is an indicator for whether the individual had been diagnosed before December of 2022, when the first cohort affected by the reform was 65 y old—an age where the ADRD incidence is still relatively low. Despite the low incidence, our findings indicate that education has a negative causal effect on ADRD incidence. Moreover, the school reform mitigated the genetic risk of developing ADRD, with large reductions in incidence even among the high genetic risk group.

The rest of the paper is structured as follows. Section 1 presents the data and describes the school reform. The main results are presented in Section 2. Section 3 investigates whether the school reform mitigated the genetic risk for ADRD. Section 4 examines some potential mechanisms for the main results. Section 5 concludes.

## 1. Materials and Methods

### 1.1. Data.

We use data from the UKB, a large, population-based study initiated by the UK National Health Service (NHS) (18). Between 2006 and 2010, invitations were mailed to 9.2 million people between the ages of 40 and 69 who were registered with the NHS and lived up to about 25 miles from one of 22 study assessment centers distributed throughout the United Kingdom (19). The UKB sample is formed by 502,363 individuals who agreed to participate.

The UKB’s Outcome Adjudication Group identified dementia outcomes using hospital records, mortality data, and (self-reported) medical history. A validation study has shown that at least 84.5% of the diagnoses coded based on this algorithm are true positives (20). These diagnoses are also associated with plasma proteins that have been implicated in ADRD (21)—see *SI Appendix*, Fig. S2. Our main dementia outcome is equal to 1 if a participant had been diagnosed by December 2022 (and 0 otherwise)^*^; at the cohort level this measure translates into cumulative incidence in December 2022.

Our working sample consists of between 45,715 to 107,200 of UKB participants, depending on our sample restrictions. The Online Appendix provides details about the construction of the working samples (*SI Appendix*, Fig. S1) and of the variables used in the analysis (*SI Appendix*, Tables S1, S2, and S3).

### 1.2. The Natural Experiment.

In 1972, England, Scotland, and Wales increased the minimum age at which students could drop out of school from 15 to 16 y of age. The reform (The 1972 Raising of School-Leaving Age Act or ROSLA) affected only students born on or after September 1, 1957, who had to stay in school until at least age 16 (“treated”). Students born on August 31, 1957, or before were still allowed to drop out at age 15 (“control”).

**Fig. 1.**
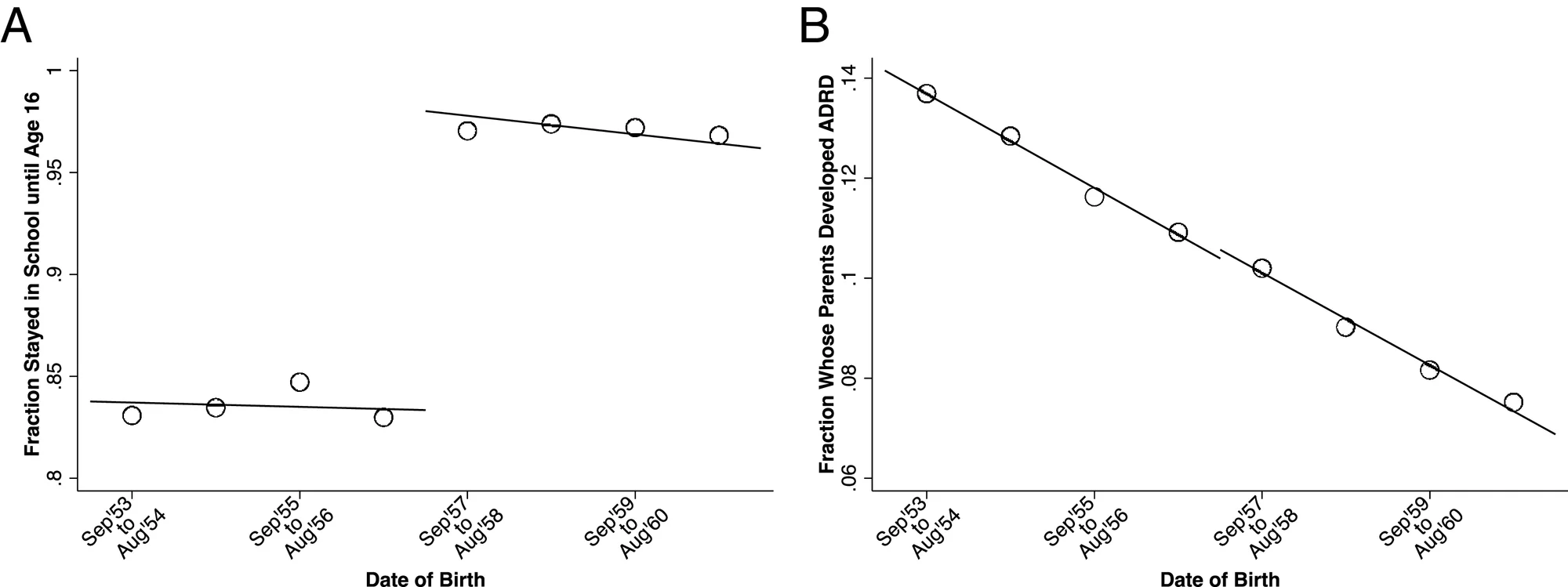
The natural experiment. Notes: The figures plot (*A*) the fraction of each birth cohort who stayed in school until age 16 and (*B*) the fraction of each birth cohort whose parents developed ADRD. The cohort born between September 1, 1957, and August 31, 1958, was the first affected by the reform. The lines show linear trends in date of birth, which are allowed to be different for those born before and after September 1, 1957. Figure (*A*) shows that the reform generated a discontinuity in the relationship between education and date of birth. Figure (*B*) shows that the parents of those born right after September 1, 1957, are as likely to have ADRD as the parents of those born right before September 1, 1957. This illustrates that the latter provides a valid counterfactual of what would have happened to the former had they not been forced to stay in school until age 16. *N* = 107,200 (*A*) and 96,738 (*B*).

Fig. 1*A* shows that the ROSLA introduced a sharp discontinuity in the relationship between education and date of birth. The markers show the fraction who stayed in school until (at least) age 16 (y-axis), separately by year of birth (x-axis). Year of birth runs from September 1 of a given year to August 31 of the following year. The lines correspond to linear trends (in exact date of birth), with different trends for those born before and after September 1, 1957, the birthdate cutoff. The graph includes individuals born within 4 y of this cutoff. The sample is further restricted to UKB participants born in England, Scotland, or Wales or who immigrated to the United Kingdom when they were 14 y of age or younger (N = 107,200).^†^

There is a large jump for the cohort born between September 1957 and August 1958, which was the first cohort required to stay in school until age 16. They were 14 percentage points more likely to stay in school until age 16 than those born right before the birthdate cutoff.

This setting provided a unique opportunity to study the causal effects of education on ADRD incidence. If, as hypothesized, there is a causal relationship between education and ADRD, then one would expect to observe a corresponding discontinuity in the relationship between ADRD and date of birth at September 1, 1957. Because individuals born right before and right after this date would have had similar ADRD incidence in the absence of the policy, any observed discontinuity could be attributed to the difference in schooling engendered by the policy.

We exploit this natural experiment using a regression discontinuity design (RDD). The RDD identifying assumption is that, in the absence of the reform, our outcome of interest would have been smooth across the September 1, 1957, threshold. Fig. 1*B* provides evidence that supports this assumption. It shows the fraction of UKB participants whose parents had been diagnosed with ADRD, separately by date of birth. Parents of those born right after September 1, 1957, were as likely to suffer from ADRD as the parents of those born right before September 1, 1957. *SI Appendix*, Fig. S4 shows that other prereform characteristics are smooth across the birthdate cutoff, including individuals’ genetic risks for ADRD.^‡^ These results strengthen our confidence that the RDD results provide unbiased estimates of the causal effects of education on the ADRD of UKB participants.

## 2. Main Results

**Fig. 2.**
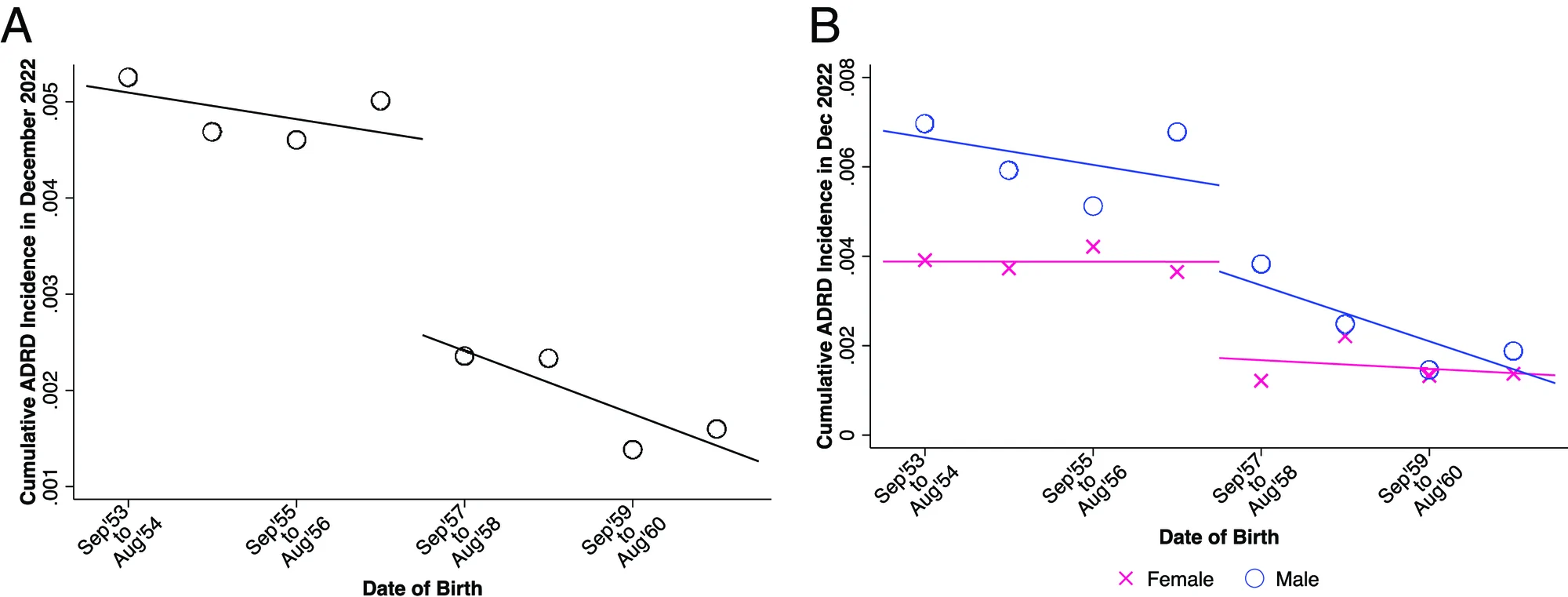
Education reduces cumulative incidence of ADRD. Notes: Figure (*A* and *B*) plot the fraction of each birth cohort diagnosed with dementia—specifically, the cumulative incidence as of December of 2022, when individuals born on September 1, 1957, were 65 y old. The cohort born between September 1, 1957, and August 31, 1958, was the first affected by the reform. The lines show linear trends in date of birth, allowed to differ for those born before and after September 1, 1957. Figure (*A*) shows a sharp decline in the cumulative incidence of ADRD for those born after September 1, 1957, illustrating the causal effects of education on ADRD. Figure (*B*), which shows separate results for women (pink) and men (blue), indicates that the effect was similar for men and women. *N* = 107,200.

Our findings indicate that education has a negative causal effect on ADRD incidence. Fig. 2*A* shows the relationship between ADRD and date of birth. There is a sharp decline in ADRD cumulative incidence among those born after September 1, 1957. Fig. 2*B* shows that there were similar declines for men and women.

Table 1 presents regressions estimates. Column (1) in the top panel estimates the first-stage:

[1] $${\mathrm{SLA}}_{i}=\alpha_{0}+\alpha_{1}{\mathrm{Post}}_{i}+\alpha_{2}{\mathrm{DoB}}_{i}+\alpha_{3}\left({\mathrm{Post}}_{i}\times {\mathrm{DoB}}_{i}\right)+u_{i}.$$

Where ${\mathrm{SLA}}_{i}$ is individual i’s school-leaving age; ${\mathrm{Post}}_{i}$ is an indicator for whether individual i was born on or after September 1, 1957; and ${\mathrm{DoB}}_{i}$ is individual i’s date of birth. Date of birth is measured in days relative to the cutoff, such that DoB=0 for someone born on September 1, 1957. The term ${\mathrm{DoB}}_{i}$ captures linear birth cohort trends in education. The term $\left({\mathrm{Post}}_{i}\times {\mathrm{DoB}}_{i}\right)$ allows these trends to be different for those born before and after September 1, 1957. We use optimal bandwidths from (22) and also report their robust bias-corrected CI. Column (1) of Table 1 confirms the graphic results shown in Fig. 1: The ROSLA led students to stay approximately 0.19 y longer in school.

**Table 1.** Regression estimates of the effect of the reform and the effect of education

	All			Dropped out at age 18 or younger		
	*School-leaving age* (1)	*ADRD (in p.p.)* (2)	*ADRD (in p.p.)* (3)	*School-leaving age* (4)	*ADRD (in p.p.)* (5)	*ADRD (in p.p.)* (6)
***Top panel***						
Born after	0.19	−0.20		0.38	−0.29	
	(0.05)	(0.08)		(0.03)	(0.12)	
	[0.103, 0.310]	[−0.397, −0.034]		[0.331, 0.453]	[−0.592, −0.046]	
School-leaving age			−1.23			−0.88
			(0.58)			(0.43)
			[−2.710, −0.181]			[−1.763, −0.154]
*Bandwidth in years*	3.0	4.1	3.9	1.7	4.1	2.6
*Observations*	80,244	108,725	105,369	25,738	60,941	38,647
*Mean of Y among those born before*	18.71	0.49	0.49	16.16	0.61	0.61
***Bottom panel***						
Born after × high risk	0.18	−0.23		0.38	−0.35	
	(0.06)	(0.12)		(0.04)	(0.17)	
	[0.086, 0.432]	[−0.525, 0.144]		[0.345, 0.560]	[−0.806, 0.108]	
Born after × low risk	0.18	−0.13		0.36	−0.17	
	(0.07)	(0.10)		(0.04)	(0.16)	
	[−0.005, 0.383]	[−0.440, 0.131]		[0.310, 0.514]	[−0.744, 0.212]	
School-leaving age × high risk			−1.38			−1.05
			(0.84)			(0.51)
School-leaving age × low risk			−0.90			−0.52
			(0.75)			(0.47)
High risk	0.06	0.15	9.16	0.00	0.06	8.69
	(0.07)	(0.12)	(21.20)	(0.04)	(0.19)	(11.30)
*P*-value, test effect high risk = effect low risk	0.965	0.476	0.670	0.653	0.448	0.439
*Bandwidth in years, high risk*	3.0	5.0	5.0	2.3	4.9	4.9
*Bandwidth in years, low risk*	3.7	4.1	4.1	1.9	4.1	4.1
*Observations*	83,162	112,752	112,752	29,306	61,708	61,708
*Mean of Y among those born before*	18.69	0.48	0.48	16.16	0.61	0.61

Notes: The dependent variables are indicated in the column headings. “Born after” is an indicator for being born on or after September 1, 1957. All regressions include linear trends in date of birth; those born before and after September 1, 1957, are allowed to have different trends. Columns (1, 2, 4, and 5) report OLS estimates. Columns (3 and 6) report two-stage least squares estimates. In columns (4 to 6), the sample is restricted to individuals who dropped out at age 18 or younger. In the top panel, optimal bandwidths are selected using the procedure of (22). Robust SE are reported in parentheses and robust bias-corrected 95% CI. In the bottom panel, “High Risk” and “Low Risk” correspond to individuals in the top and bottom halves of the distribution of the genetic risk measure. In columns (1, 2, 4, and 5) of the bottom panel, the *P*-value corresponds to a test of whether the coefficients on Born after × High Risk and Born after × Low Risk are equal. In columns (3 and 6) of the bottom panel, the *P*-value corresponds to a test of whether the coefficients on School-Leaving Age × High Risk and School-Leaving Age × Low Risk are equal. In the bottom panel, optimal bandwidths are selected using the procedure of (23). See Online *SI Appendix*, *Appendix C* for more technical details regarding regression specifications.

The reduced-form is estimated in column (2) of the top panel:

[2] $${\mathrm{ADRD}}_{i}=\beta_{0}+\beta_{1}{\mathrm{Post}}_{i}+\beta_{2}{\mathrm{DoB}}_{i}+\beta_{3}\left({\mathrm{Post}}_{i}\times {\mathrm{DoB}}_{i}\right)+v_{i},$$

where ${\mathrm{ADRD}}_{i}$ is an indicator for whether individual i had been diagnosed with dementia by December of 2022. We also use (22) to select optimal bandwidths (1,217 d) and estimate CI and *P*-values. We find that those born after September 1, 1957, were 0.2 percentage points less likely to have been diagnosed with ADRD.

Column (3) in the top panel estimates a two-stage least squares model:

[3] $${\mathrm{ADRD}}_{i}=\gamma_{0}+\gamma_{1}{\mathrm{SLA}}_{i}+\gamma_{2}{\mathrm{DoB}}_{i}+\gamma_{3}\left({\mathrm{Post}}_{i}\times {\mathrm{DoB}}_{i}\right)+\varepsilon_{i},$$

where ${\mathrm{Post}}_{i}$ is used to instrument for ${\mathrm{SLA}}_{i}$. *P*-values and CI for two-stage least squares estimates are based on an Anderson-Rubin test (*SI Appendix*, *Appendix A*), which have been shown to have better properties than two-stage least squares *t* tests (24). We find that staying in school 1 y longer reduced ADRD cumulative incidence by 1.2 percentage points (*P*-value 0.020).

Columns (4 to 6) re-estimate these same equations using a different sample, namely those who left school at age 18 or younger. This sample restriction yields tighter CI because of the stronger first-stage. It yields unbiased 2SLS estimates under the assumption that the school reform did not affect the fraction who stayed in school until at least age 19—*SI Appendix*, Fig. S5 shows that this was the case.

Column (6) shows our preferred 2SLS specification.^§^ For those who left school at age 18 or younger, staying in school 1 y longer reduced ADRD cumulative incidence by approximately 0.88 percentage point (*P*-value 0.022). This is a surprisingly large point estimate given the low baseline incidence in this young cohort (0.61), but we note that the CI are also wide, even in the preferred, restricted sample (95% CI: [−1.763, −0.154]). In other words, we can reject reductions smaller than 24% of the baseline incidence.^¶^ While our data and methods allow us to credibly estimate the direction of the causal effect of education on dementia diagnosis, we are not powered to estimate its precise magnitude. Instead, we focus on the magnitudes we can reject given our CI. Overall, we see our results as providing strong evidence that increased education has a protective effect against dementia, but we caution against overinterpreting the magnitude of our point estimates.

## 3. Genetics and Heterogenous Effects

A common misperception is that the influence of genetics on a disease such as ADRD cannot be altered by environmental interventions—presumably because it is assumed that this influence reflects a biological, immutable relationship. However, because genetic effects can operate through environmental channels, intervening on those channels could in principle influence genetic effects (25, 26). In this section, we investigate whether education can mitigate the genetic risk for ADRD (13). We begin by discussing how we measure this risk.

As a measure of one’s genetic risk factor for developing ADRD, we constructed an ADRD polygenic index (PGI). Our PGI was constructed using two different types of genetic variation. The first type are Single Nucleotide Polymorphisms (SNPs). The second type of variation are the APOE variants. Individuals with some APOE variants are at much greater risk of developing ADRD—e.g., having two copies of APOE4 is associated with a 10-fold higher risk. The weights for the SNP genotypes are based on the largest Genome Wide Association Study (GWAS)^#^ for Alzheimer’s disease that omits the UK Biobank (27).^||^ We use a simple model to estimate the weights for the pair of APOE variants, $\lambda_{k}$ (see *SI Appendix*, *Appendix B* for more details on how we constructed the ADRD PGI).^**^^,^^††^ Because APOE has such a large effect on ADRD incidence, much of the variation in our PGI was driven by APOE (58% of those in the top half had at least one E4 allele, while only 0.02% of those in the bottom half did). Importantly, our PGI is fixed at conception and therefore cannot be affected by the school reform.

### 3.1. Graphical Results.

**Fig. 3.**
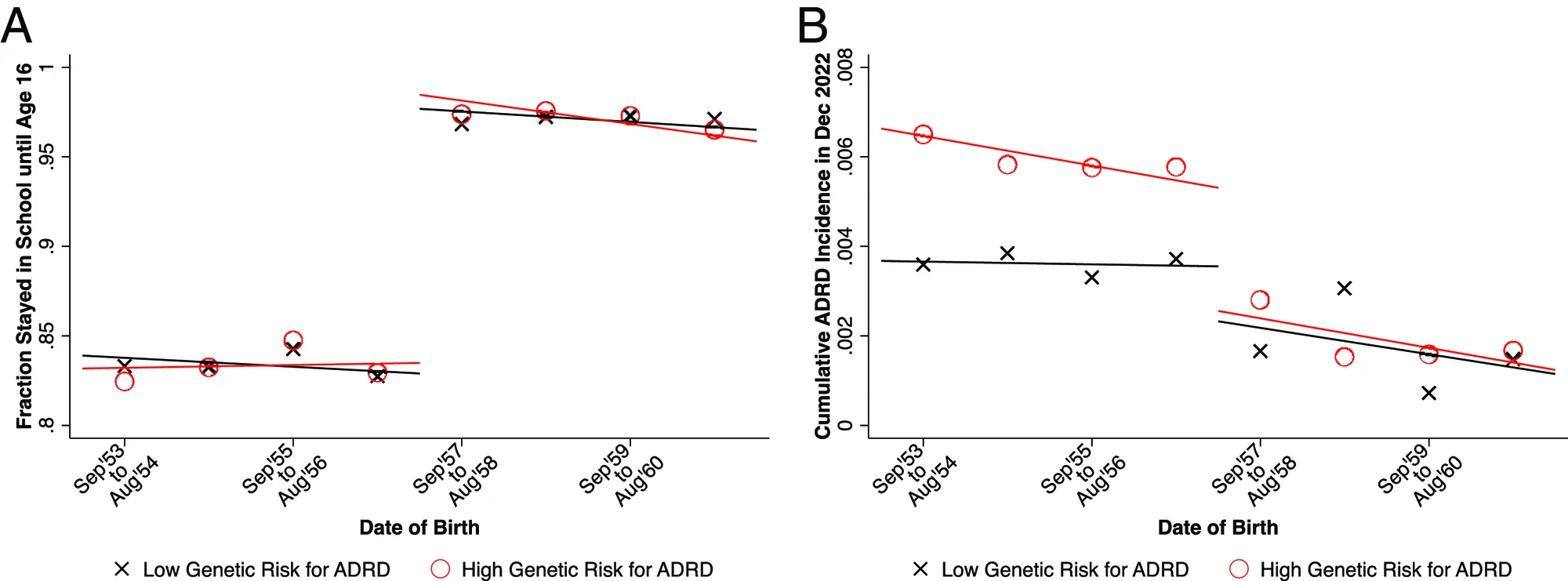
Reduction in ADRD cumulative incidence is larger for those with greater ADRD genetic risk. Notes: We measured one’s genetic risk of developing ADRD by constructing an ADRD polygenic index that includes both a polygenic component and a person’s APOE genotype. Separate results are shown for those in the bottom half (black) and in the top half (red) of the distribution of this index. The figures plot (*A*) the fraction of each birth cohort who stayed in school until age 16 and (*B*) the fraction of each birth cohort diagnosed with ADRD. The cohort born between September 1, 1957, and August 31, 1958, was the first affected by the reform. The lines show linear trends in date of birth, which are allowed to be different for those born before and after September 1, 1957. Figure (*A*) shows that the reform affected the education of the two groups similarly. Figure (*B*) shows that it had a larger effect on the prevalence rate of those in the top half. *N* = 98,778.

Fig. 3 investigates how two groups—those in the bottom and top halves of the distribution of this PGI—were affected by the school reform. The black markers and black line show effects on those in the bottom half, who have a lower genetic risk of developing ADRD. The red markers and red line show effects on those in the top half, who have a higher genetic risk of developing ADRD.

Fig. 3*A* shows that the policy affected the education of the two groups similarly. In both cases, those born right after September 1, 1957, were 14 percentage points more likely to stay in school until age 16 than those born right before. Thus, any differences in the effect of the policy on ADRD across the two groups cannot be explained by differences in how much the policy impacted the education of these two groups.^‡‡^

Fig. 3*B* shows the ADRD cumulative incidence for the two groups, separately by year of birth. For the cohorts born before September 1, 1957, there is a gap between the red and black lines, reflecting the larger ADRD incidence among those in the top half of the distribution of the PGI. If risk due to genetic factors were immutable, the gap between the red and black lines would remain for those born after September 1, 1957.

The reform caused, however, a greater reduction in the cumulative incidence of ADRD of those in the top half, virtually closing the gap between the two groups. It reduced the difference in the cumulative incidence between the two groups from 0.18 percentage points to 0.02 percentage points.

### 3.2. Regression Estimates.

We further investigate these results in the bottom panel of Table 1. We re-estimate the same specifications as in the table’s top panel, allowing the effects now to vary with one’s genetic risk factor. In the bottom panel of Table 1, we use an extension of (22)’s method that allows for heterogeneous treatment effects (23) to select the optimal bandwidth and to compute bias-corrected CI for reduced form estimates. Such methods are currently not available for 2SLS, therefore we report conventional robust SE for the 2SLS estimates in the table. Columns (1 and 2) estimate:

[4] $$\begin{array}{c} Y_{i}=\delta_{0}+\delta_{1}\left({\mathrm{Post}}_{i}\times G_{i}\right)+\delta_{2}{\mathrm{Post}}_{i}+\delta_{3}G_{i}+\delta_{4}{\mathrm{DoB}}_{i}\\ \;\;+\delta_{5}\left({\mathrm{Post}}_{i}\times {\mathrm{DoB}}_{i}\right)+\epsilon_{i}, \end{array}$$

where $Y_{i}$ is ${\mathrm{SLA}}_{i}$ in column (1) and ${\mathrm{ADRD}}_{i}$ in column (2); and $G_{i}$ is a dummy for whether individual i is in the top or bottom halves of the distribution of the genetic risk measure. Column (3) in the bottom panel estimates a two-stage least squares model:

[5] $$\begin{array}{c} {\mathrm{ADRD}}_{i}=\eta_{0}+\eta_{1}\left({\mathrm{SLA}}_{i}\times G_{i}\right)+\eta_{2}{\mathrm{SLA}}_{i}+\eta_{3}G_{i}+\eta_{4}{\mathrm{DoB}}_{i}\\ \;\;+\eta_{5}\left({\mathrm{Post}}_{i}\times {\mathrm{DoB}}_{i}\right)+\zeta_{i}, \end{array}$$

where again ${\mathrm{Post}}_{i}$ is used to instrument for ${\mathrm{SLA}}_{i}$ and $\left({\mathrm{Post}}_{i}\times G_{i}\right)$ is used to instrument for $\left({\mathrm{SLA}}_{i}\times G_{i}\right)$. Columns (4 to 6) re-estimate columns (1 to 3), restricting the sample to those who dropped out at age 18 or younger.

Columns (2 and 5), which are consistent with Fig. 3*B*, show that the reform mitigated the genetic risks of developing ADRD. While we find that both the high and low risk groups see a reduction in ADRD incidence, this reduction is only significant for the high-risk group. That said, we cannot reject the hypothesis that the effect of the reform on ADRD is the same for both groups, potentially due to low power on these binary interaction estimates. When we turn to our preferred 2SLS estimates in column (6), we see similar results: the drop in ADRD is about twice as large for those with a high genetic risk vs. those with a low genetic risk and it is only significantly different from zero for the high-risk group. These are encouraging results, suggesting that ADRD incidence from genetic factors can be mitigated, and that changes in environmental circumstances, such as education, can reduce ADRD incidence even in a population with high incidence due to their genes.^§§^

In principle, the relationship between dementia and the PGI, as captured by $\delta_{3}$ and $\eta_{3}$ in Eqs. **4** and **5**, could be confounded by environmental factors. After all, the PGI is constructed based on weights that reflect associations between ADRD and genetic markers. However, due to the properties of Mendelian inheritance, the genes that one inherits are randomly assigned conditional on the genes of the one’s parents (28–30). In *SI Appendix*, *Appendix B* we leverage this family-level variation to obtain an unbiased estimate of the causal effect of the ADRD PGI, documenting that there is little environmental confounding in this context and providing evidence that the estimates in this section reflect causal genetic effects.

## 4. Potential Mechanisms

There are several ways through which the ROSLA reform may have reduced ADRD incidence. For example, those affected may have acquired additional skills or qualifications that gave them access to more cognitively stimulating jobs, thereby building their cognitive reserves. The reform may also have increased their income, making a healthy lifestyle more affordable, or placed them in social environments with healthier peers, encouraging the adoption of healthy habits.

Unfortunately, because we have just one source of exogenous variation, we cannot quantify the relative importance of different mechanisms. Nevertheless, we can still investigate whether a proxy for a given channel was impacted by the school reform—a necessary condition for the potential mechanism to mediate the relationship between education and ADRD.

The UK Biobank contains rich data on health conditions considered risk factors for ADRD, including objective measures of obesity, hypertension, and diabetes (a HbA1c Test). Stroke, infarction, and chronic obstructive pulmonary disease (COPD) were identified from a combination of self-reports, hospitalization data, and death records and are measured as of December of 2022. All the other outcomes were measured at baseline when the analyzing sample was between 45 and 56 y of age.

**Fig. 4.**
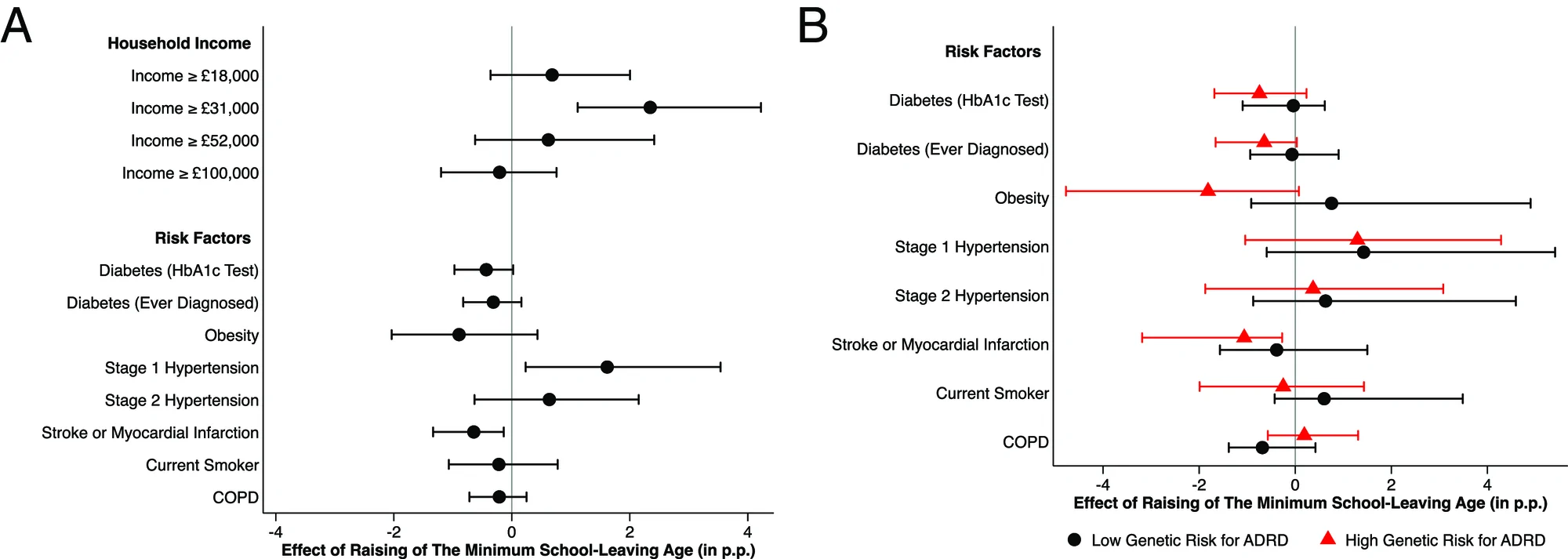
Potential mediators: income and risk factors. Notes: The figure examines some potential channels through which the additional schooling may have affected ADRD incidence. It shows intent-to-treat estimates of the effect of the policy on the outcomes listed in the rows. Estimates are in percentage point units. While (*A*) shows estimates for the entire sample, (*B*) estimates separate effects on those in the bottom half (black) and on those in the top half (red) of the distribution of the ADRD genetic risk index. The brackets represent robust bias-corrected 95% CI (22, 31), while the point estimates are uncorrected. Because the bias correction is applied only to the CI, the point estimates (markers) are not necessarily centered within the brackets. In panel (*A*), optimal bandwidths are selected using the procedure of (22). In panel (*B*), optimal bandwidths are selected using the procedure of (23). We apply triangular kernel weights, which place greater weight on individuals born closer to the cutoff.

Fig. 4*A* shows intent-to-treat estimates of the effect of the policy on household income and on ADRD risk factors. Each row corresponds to a separate outcome (all effects are measured in percentage points). The markers show point estimates while the brackets show 95% CI.

Those affected by the school reform were 2.3 percentage points more likely to have an income of £31,000 or more (*P*-value 0.001); 0.43 percentage points less likely to have diabetes (*P*-value 0.062); and 0.64 percentage points less likely to have had a stroke or myocardial infarction (*P*-value 0.016). Interestingly, they were also more likely to develop stage 1 hypertension, in line with what was reported in (32). These results are consistent with other papers using the UKB data that show that the ROSLA had positive effects on health and SES (33, 34).^¶¶^

Fig. 4*B* re-estimates the effects on these risk factors, separately for those in the bottom and top halves of the distribution of the ADRD genetic risk factor. We see larger reductions in diabetes and obesity for the high genetic risk group, but these are not statistically significant (*P*-value = 0.060 and 0.058 for ever diagnosed with diabetes and obesity respectively). The reduction in stroke and myocardial infarction is also driven by the high-risk group and significant (coefficient −1.061, *P*-value 0.020). The previous section showed that the reduction in ADRD incidence was larger for the high-genetic-risk group. The effects of the school reform on some of these mechanism-proxies (i.e., diabetes, stroke) were also larger for the high genetic risk group, providing additional suggestive evidence that these mechanisms are at play.

## 5. Conclusion

We found that the additional schooling induced by a natural experiment in the United Kingdom in the early 1970s subsequently reduced the cumulative incidence of ADRD in affected cohorts. These results suggest that ADRD incidence can be modified by social policy, a welcome finding given the expected exponential growth in cases of dementia and their large costs. Encouragingly, we also found that the school reform mitigated the genetic risk of developing ADRD—rejecting the common perception that the influence of genetics on diseases such as ADRD cannot be altered by environmental interventions.

Scholars have noted the need for high-quality evidence on which factors can reduce ADRD and delay its onset (9, 35). Such evidence is paramount for designing policies that can prevent and effectively treat dementia. Characteristics that are highly associated with ADRD risk have been deemed as risk factors for dementia, but there is limited evidence as to whether these characteristics causally affect ADRD (35, 36).

Education is a case in point. Lower education has been singled out as a major risk factor for ADRD, but there is limited evidence on the causal effect of education on dementia. While previous studies found evidence that education causally improves cognition and slows cognitive decline (37–41), we are aware of only one other paper that studied the causal relationship between education and ADRD diagnosis using a natural experiment combined with dementia measures derived from administrative health records. Seblova et al. (42) studied the effects of a compulsory schooling age reform in Sweden, exploiting temporal variation in when school districts implemented the policy: early-adopters implemented the reform as early as 1936 while late-adopters implemented it as late as 1953. Inpatient and death registries were used to measure dementia diagnosis for over 1.3 million individuals. The study found no effect of the reform on ADRD diagnosis. It also found limited effects on socioeconomic outcomes such as income.

We use the ROSLA as a natural experiment that lends itself to a compelling regression discontinuity design (33, 34). We document a causal relationship between education and ADRD diagnosis and show that education can mitigate genetic risk for ADRD.

To translate this evidence into policy-relevant interventions, it is important to understand the channels through which education affects ADRD incidence. While the reform in Sweden studied by Seblova et al. (42) seems to have had limited, if any, effects on socioeconomic outcomes, it is clear that the 1972 ROSLA improved socioeconomic status—suggesting that this may be one of the mechanisms through which education reduces ADRD incidence. The ROSLA may have also improved some health outcomes, reducing diabetes, obesity, stroke, and infarction, but much remains to be understood.

Our 2SLS point estimate implies that staying in school a year longer reduces ADRD cumulative incidence by approximately 0.88 percentage points. There are reasons to expect a large effect in this setting: by keeping students in school until age 16, the ROSLA might have made an especially consequential change in their life trajectories. Barcellos et al. (43) find that the ROSLA affected schooling qualifications, income, and occupational status, benefits that might have compounded over the roughly five decades between the reform onset and the measurement of dementia incidence. Nevertheless, our point estimate is larger than the ADRD incidence in both the overall population and among reform compliers, which is implausible.

One potential concern is that our estimates could be inflated by differential diagnosis: if more educated individuals are less likely to be diagnosed with dementia for a given level of underlying pathology, we would overstate the effect of education. The direction of this bias is theoretically ambiguous—more educated individuals may compensate for pathology and evade diagnosis, but they also have better access to healthcare, which could make diagnosis more likely. Empirically, studies comparing research-based clinical assessments to diagnoses recorded in administrative data find that, conditional on clinical dementia, more educated individuals are at least as likely to be diagnosed (44–48). We therefore view differential diagnosis as an unlikely explanation for the large magnitude.

Instead, we believe that sampling variation is the most plausible explanation. Although our sample is large, ADRD remains a rare outcome in these relatively young cohorts, and regression discontinuity designs require very large samples to achieve precise estimates. As a result, we can credibly estimate the direction of the causal effect of education, but not its precise magnitude. Our CI is wide and includes a range of economically meaningful effects. However, our 95% CI does exclude reductions of less than 0.15 percentage points, which is a quarter of the baseline ADRD incidence. We view this paper as providing important causal evidence that education has a protective effect against dementia, but we advise against placing too much weight on the point estimate itself. Instead, we suggest focusing on the magnitudes that can be rejected given our CI.

This study has other limitations. First, our results are specific to the natural experiment we studied and to the lower-education population affected by the ROSLA. Admittedly, the effects of education on ADRD may vary with the level of education and across contexts. Second, our results are specific to UKB participants, who tend to have higher socioeconomic status and better health than the general population (49). Moreover, the great majority of them are of European ancestries and results might not apply to more diverse populations. Finally, the cohort studied in this paper is relatively young; individuals born on September 1, 1957, were 65 y old in 2022. Some may worry that dementia cases diagnosed before age 65 are primarily caused by rare genetic variants that operate through biological pathways that would not be modifiable by environmental interventions. However, such variants account for only about 10% of ADRD cases diagnosed before age 65 (50, 51). Another concern is that early-onset and late-onset dementia may have distinct etiologies, raising questions about the generalizability of our findings to older populations. Yet some researchers have questioned whether these forms of dementia are truly etiologically distinct (52), and recent evidence from the UK Biobank shows that many of the same risk factors predict ADRD diagnosed both before and after age 65 (53).

Although the ADRD prevalence in our sample was lower than 1%, we were still able to identify a clear reduction in ADRD incidence due to education. Follow-up with this cohort as ADRD prevalence increases will be important.

## Supplementary Material

Appendix 01 (PDF)

## Data Availability

The UK Biobank data used in this study are available to eligible researchers through an application to UK Biobank. Researchers must meet UK Biobank’s eligibility requirements and obtain approval for a health-related research project that is in the public interest. Information on eligibility, access requirements, and the application process is available from UK Biobank (54).

## Footnotes

^*^If the individual had died before December 2022 from non-ADRD related causes, we coded this observation as a 0. We show in *SI Appendix*, Table S4 that this coding choice does not drive our results. First, we estimate a multinomial logit model that distinguishes between developing ADRD; dying from a non-ADRD related cause; or being alive without ADRD. We then estimate a logit model that does not distinguish between the last two. The two specifications yield identical estimates, which lead us to conclude that our main findings are not driven by effects of the school reform on mortality.

^†^We excluded from the sample participants for whom data on the age at which they left full-time education were missing.

^‡^*SI Appendix*, Fig. S3 shows the results from a McCrary Test.

^§^*SI Appendix*, Table S5 shows that results in Table 1 are similar if we use a logit model instead of a linear probability model.

^¶^*SI Appendix*, Fig. S6 shows how the reduced form effects vary as the sample ages, and that the discontinuity becomes clearer as we add the last 4 y of linked registry data. It will be important to keep following this cohort and to re-estimate the effects of the reform as they age and as ADRD incidence in the sample increases.

^#^A GWAS scans the entire genome and estimates associations between individual genotypes and an outcome of interest. Specifically, a GWAS is a series of regressions of some outcome onto the genotype of each SNP, one at a time, and a set of covariates which normally include sex, age, and the first several principal components of the genetic data.

^||^There are several methods for producing PGI weights from GWAS coefficients, but each of them transforms the GWAS coefficients in a way that is meant to account for the correlation structure that exists in the genome. The GWAS associations corresponding to the HapMap3 SNPs were transformed to account for linkage disequilibrium (LD) using the SBayesR method (55), and the EUR subsample of the 1,000 Genomes Project data as a reference panel (56). Due to the outsized effect of the APOE region of ADRD risk, we omitted the region on chromosome 19 from basepair position 44.4-46.5 Mb (57).

^**^To avoid overfitting, these weights were estimated using a set of UK Biobank participants outside our optimal bandwidth.

^††^Following the standard practice, we restrict the sample to individuals whose genetic principal components cluster with the EUR subsample of a genomics reference panel, the 1,000 Genomes Project. Due to Euro-centric bias in data collection, most currently published GWASs, including (27), are based on samples with “European” ancestries. As a result, PGIs based on currently available GWAS coefficients are substantially more predictive in European-ancestry samples (58) and may not generalize to groups with African, Asian, Hispanic, or other non-European ancestries. This sample restriction reduces the correlation between a person’s genotypes and their environment—such a correlation could produce omitted variable bias in estimates of the effect of genetic variables, such as PGIs, on various outcomes.

^‡‡^Note that the two groups—those with low and high genetic risks—are allowed to have different trends in Fig. 3.

^§§^*SI Appendix*, Table S5 reports logit estimates corresponding to the results in columns (2 and 5) of the bottom panel of Table 1. Both types of model yield similar conclusions.

^¶¶^Nevertheless, it is important to note that there is currently no consensus on whether education has a causal effect in health in general (59).
