# Supplementary material for: Education reduces dementia incidence and mitigates genetic risk
Source: Proc Natl Acad Sci U S A. 2026 Oct 23;123(43):e2529506123. doi: 10.1073/pnas.2529506123 (PMC13715751; doi:10.1073/pnas.2529506123)
Supplement: Supplementary file 1 [file pnas.2529506123.sapp.pdf]

Supporting Information for

Education Reduces Dementia Incidence and Mitigates Genetic Risk

Authors: Silvia H. Barcellos[1,2,3]* Leandro S. Carvalho[3,4], Kenneth M. Langa[5,6], Sneha Nimmagadda[4], and Patrick Turley[3,4]

Affiliations:
[1] La Follette School of Public Affairs, University of Wisconsin-Madison; Madison, WI, USA.
[2] Department of Population Health Sciences, University of Wisconsin-Madison, Madison, WI, USA.
[3] Center for Economic and Social Research, University of Southern California; Los Angeles, CA, USA.
[4] Department of Economics, University of Southern California; Los Angeles, CA, USA.
[5] Department of Internal Medicine, University of Michigan; Ann Arbor, MI, USA.
[6] Institute for Social Research, University of Michigan; Ann Arbor, MI, USA.
*Corresponding author.
Email: barcellos@wisc.edu
Address: 305 Observatory Hill Office Building, 1225 Observatory Drive, Madison, WI 53706.

This PDF file includes:

Supporting text
Figures S1 to S6
Tables S1 to S6
Appendix A, B and C
SI References
Replication Package

TABLE OF CONTENTS

	Page
Appendix Figure S1. Sample	3
Appendix Table S1. Data	4
Appendix Table S2. Clinical Codes Used to Ascertain ADRD	5
Appendix Table S3. Summary Statistics	6
Appendix Figure S2. Dementia Outcome is Associated with Plasma Proteins	7
Appendix Figure S3. McCrary Test	8
Appendix Figure S4. Balance Test	9
Appendix Figure S5. Reform Had No Effect on Fraction Staying in School Past Age 18	10
Appendix Figure S6. Effect on Cumulative Incidence at Different Ages	11
Appendix Table S4. Mortality and Selection	13
Appendix Table S5. Comparison Linear Probability versus Logit Model (Homogeneous)	14
Appendix Table S6. Comparison Linear Probability versus Logit Model (Heterogeneous)	14
Appendix A: The Anderson-Rubin Test and Confidence Intervals	15
Appendix B: Measuring genetic risk of ADRD	16
Appendix C: Table 1 extended notes	21
References	22
Replication Package	23

Appendix Figure S1. Sample. The figure shows the different sample restrictions, the size of the resulting samples, and the analyses in which these samples were used. The UK Biobank (UKB) sample is formed by 502,363 individuals. We made the following sample restrictions. First, we restricted the sample to UKB participants born in England, Scotland, or Wales or who immigrated to the UK when they were 14 years of age or younger. Second, we excluded from the sample participants for whom data on the age at which they left full-time education were missing. Third, we restricted the sample according to the optimal bandwidth determined by Calonico et al. (1)'s optimal bandwidth selection procedure. In columns (4) to (6) of Table 1, we further restricted the sample to UKB participants who dropped out at age 18 or younger. In the analyses using genetic data, the sample was restricted to individuals who had been genotyped and who were of European genetic ancestries.

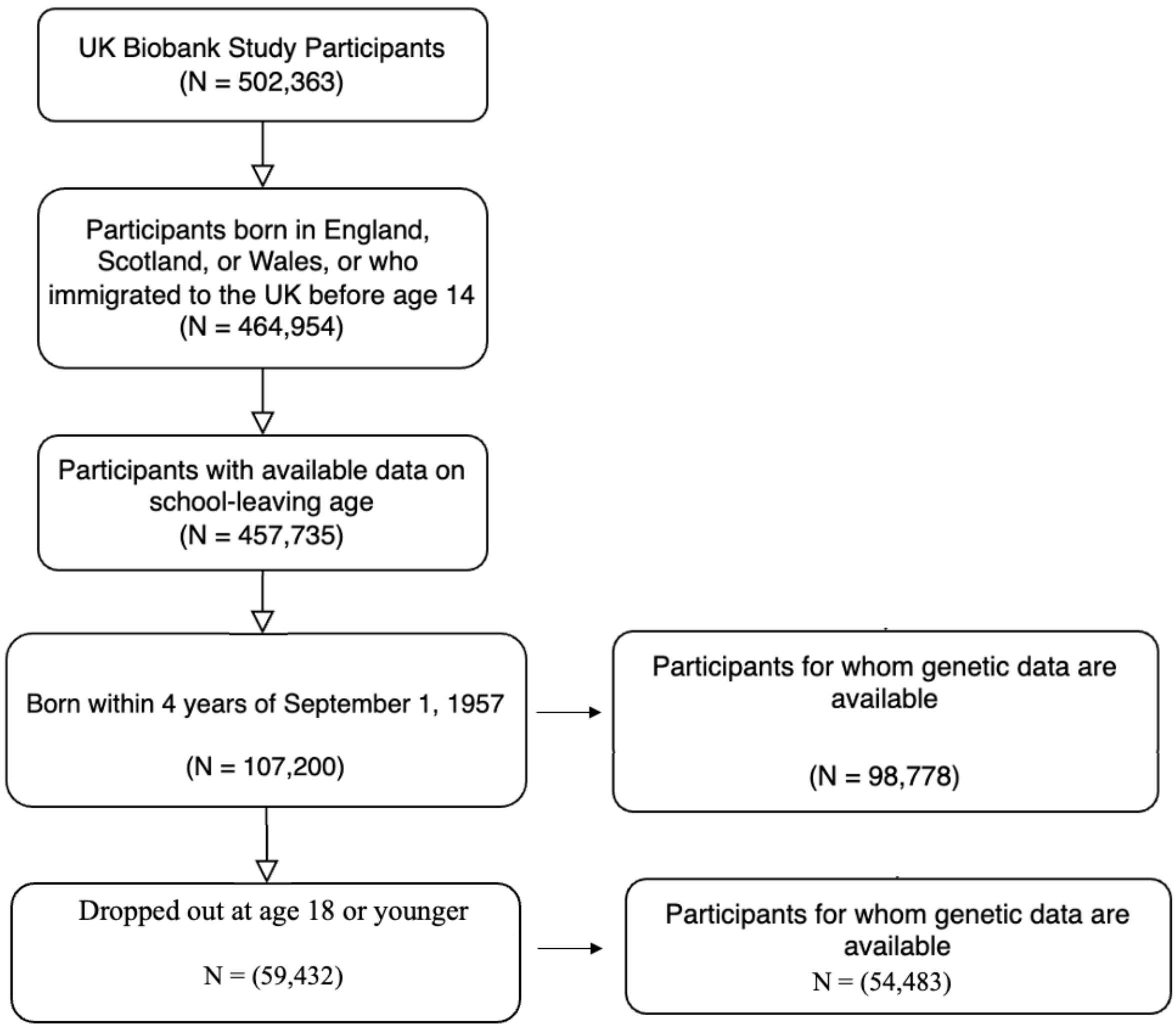

Appendix Table S1 gives details about the data. **Appendix Table S2** gives specific details about the main outcome of interest, dementia status. **Appendix Table S3** shows summary statistics.

Appendix Table S1. Data. The table shows the UK Biobank fields used to construct the data used in the main analyses. The UK Biobank Showcase gives more information about each field (see tab Notes and tab Resources). For survey questions, the table also shows the wording of the questions. Participants who answered that they had a college degree were not asked about their school-leaving ages. We assigned a school-leaving age of 22 to college graduates.

	UKB Field	Notes and *Survey Questions*
**Registry Data**		
Gender	31	Updated by participant if needed
Date of Birth	33	Updated by participant if needed
**Survey Questions**		
Educational Qualifications	6138	*Which of the following qualifications do you have?*
School-Leaving Age	845	*At what age did you complete your continuous full time education?*
Parents Developed ADRD	20107, 20110	*Has/did your father/mother ever suffer from Alzheimer's disease/dementia?*
Income Greater than £18,000	738	*What is the average total income before tax received by your HOUSEHOLD?*
Income Greater than £31,000	738	*What is the average total income before tax received by your HOUSEHOLD?*
Income Greater than £52,000	738	*What is the average total income before tax received by your HOUSEHOLD?*
Income Greater than £100,000	738	*What is the average total income before tax received by your HOUSEHOLD?*
Diabetes (Ever Diagnosed)	2443	*Has a doctor ever told you that you have diabetes?*
Current Smoker	1239	*Do you smoke tobacco now?*
**Algorithmically-Defined Outcomes**		
ADRD	42018	See UK Biobank 2022 Algorithmically-Defined Outcomes
Myocardian Infarction	42000	https://biobank.ndph.ox.ac.uk/showcase/ukb/docs/alg_outcome_main.pdf
Stroke	42006	
COPD	42016	
**Physical Measures**		
Obesity	21001, 23104	Average BMI from anthropometrics and BMI from impedance measurement
Stage 1 Hypertension	93, 94, 4079, 4080	Systolic ≥ 130 or Diastolic ≥ 80
Stage 2 Hypertension	93, 94, 4079, 4080	Systolic ≥ 140 or Diastolic ≥ 90
**Biomarker**		
Diabetes (HbA1c Test)	30750	HbA1c ≥ 6.5 https://biobank.ndph.ox.ac.uk/showcase/ukb/docs/serum_hb1ac.pdf

The UK Biobank Outcome Adjudication Group, in conjuction with clinical experts, developed algorithms that ascertain a given health outcome – e.g., ADRD – based on a list of clinical codes. For each individual, the algorithm takes the earliest recorded date in which one of these predefined codes show up in either hospital admission records or in death certificate records. Appendix Table S2 shows the ICD-9 and ICD-10 codes used to ascertain ADRD. In some cases, ADRD was ascertained from the participant's self-report of an ADRD diagnosis, in which case field 20008 is used to identify the date of diagnosis. Less than 2% of the dementia diagnoses in our data are ascertained from self-reports. Approximately 94% of the dementia diagnoses in our data come from hospital admission records. The reader is referred to the document "Algorithmically-defined outcomes" put out by the UK Biobank in January of 2022 (Version 2.0).

Appendix Table S2. Clinical Codes Used to Ascertain ADRD. The table shows the ICD 9 and ICD 10 codes used to ascertain ADRD status of UK Biobank participants.

ICD 10 Code	ICD 10 Text
A81.0	Sporadic Creutzfeldt-Jakob disease
F00	Dementia in Alzheimer's disease
F00.0	Dementia in Alzheimer's disease with early onset
F00.1	Dementia in Alzheimer's disease with late onset
F00.2	Dementia in Alzheimer's disease, atypical or mixed type
F00.9	Dementia in Alzheimer's disease, unspecified
F01	Vascular dementia
F01.0	Vascular dementia of acute onset
F01.1	Multi-infarct dementia
F01.2	Subcortical vascular dementia
F01.3	Mixed cortical and sub-cortical vascular dementia
F01.8	Other vascular dementia
F01.9	Vascular dementia, unspecified
F02	Dementia in other diseases classified elsewhere
F02.0	Dementia in Picks disease
F02.1	Dementia in Creutzfeldt-Jacob disease
F02.2	Dementia in Huntington’s disease
F02.3	Dementia in Parkinson’s disease
F02.4	Dementia in HIV disease
F02.8	Dementia in other specified diseases classified elsewhere
F03	Unspecified dementia
F05.1	Delirium superimposed on dementia
F10.6	Mental and behavioural disorders due to use of alcohol - amnesic syndrome
G30	Alzheimer’s disease
G30.0	Alzheimer’s disease with early onset
G30.1	Alzheimer’s disease with late onset
G30.8	Other Alzheimer's disease
G30.9	Alzheimer's disease unspecified
G31.0	Circumscribed brain atrophy
G31.1	Senile degeneration of brain
G31.8	Other specified degenerative diseases of nervous system
I67.3	Binswanger’s disease
**ICD 9 Code**	**ICD 9 Text**
290.2	Senile dementia, depressed or paranoid type
290.3	Senile dementia with acute confusional state
290.4	Arteriosclerotic dementia
291.2	Other alcoholic dementia
294.1	Dementia in other conditions classified elsewhere
331	Alzheimer's disease
331.1	Pick's disease
331.2	Senile degeneration of brain
331.5	Creutzfeldt-Jakob disease

Appendix Table S3. Summary Statistics. This table shows summary statistics of the variables used to produce the results shown in the paper. The sample includes those born between September 1, 1953 and August 1, 1962 (i.e., within 4 years of September 1, 1957).

	**All**			**Dropped Out at Age 18 or Younger**		
	N	*Mean*	*SD*	*N*	*Mean*	*SD*
	(1)	(2)	(3)	(4)	(5)	(6)
Male	107,200	0.44	0.50	59,342	0.43	0.50
Year of Birth	107,200	1957	2.33	59,342	1957	2.33
Born after September 1, 1957	107,200	0.47	0.50	59,342	0.47	0.50
Stayed in School until Age 16	107,200	0.90	0.30	59,342	0.82	0.39
School-Leaving Age	107,200	18.79	2.97	59,342	16.32	1.05
Parents Developed ADRD	96,738	0.11	0.31	52,316	0.10	0.30
Diagnosed with ADRD	107,200	0.004	0.06	59,342	0.004	0.06
Age at Time of ADRD Diagnosis	375	61.32	5.17	251	61.21	5.38
Genetic Risk for ADRD	98,778	0.00	1.00	54,843	0.00	1.00
High Genetic Risk for ADRD	98,778	0.50	0.50	54,843	0.50	0.50
Income Greater than £18,000	96,292	0.87	0.34	51,504	0.82	0.39
Income Greater than £31,000	96,292	0.68	0.47	51,504	0.57	0.50
Income Greater than £52,000	96,292	0.38	0.48	51,504	0.25	0.43
Income Greater than £100,000	96,292	0.08	0.27	51,504	0.03	0.17
Diabetes (HbA1c Test)	100,116	0.03	0.16	55,265	0.03	0.18
Diabetes (Ever Diagnosed)	106,958	0.03	0.18	59,167	0.04	0.19
Obesity	106,763	0.25	0.43	59,080	0.29	0.45
Stage 1 Hypertension	107,034	0.68	0.47	59,237	0.70	0.46
Stage 2 Hypertension	107,034	0.37	0.48	59,237	0.40	0.49
Stroke or Myocardial Infarction	107,200	0.05	0.23	59,342	0.06	0.24
Current Smoker	107,136	0.12	0.33	59,302	0.15	0.36
COPD	107,200	0.03	0.18	59,342	0.05	0.21

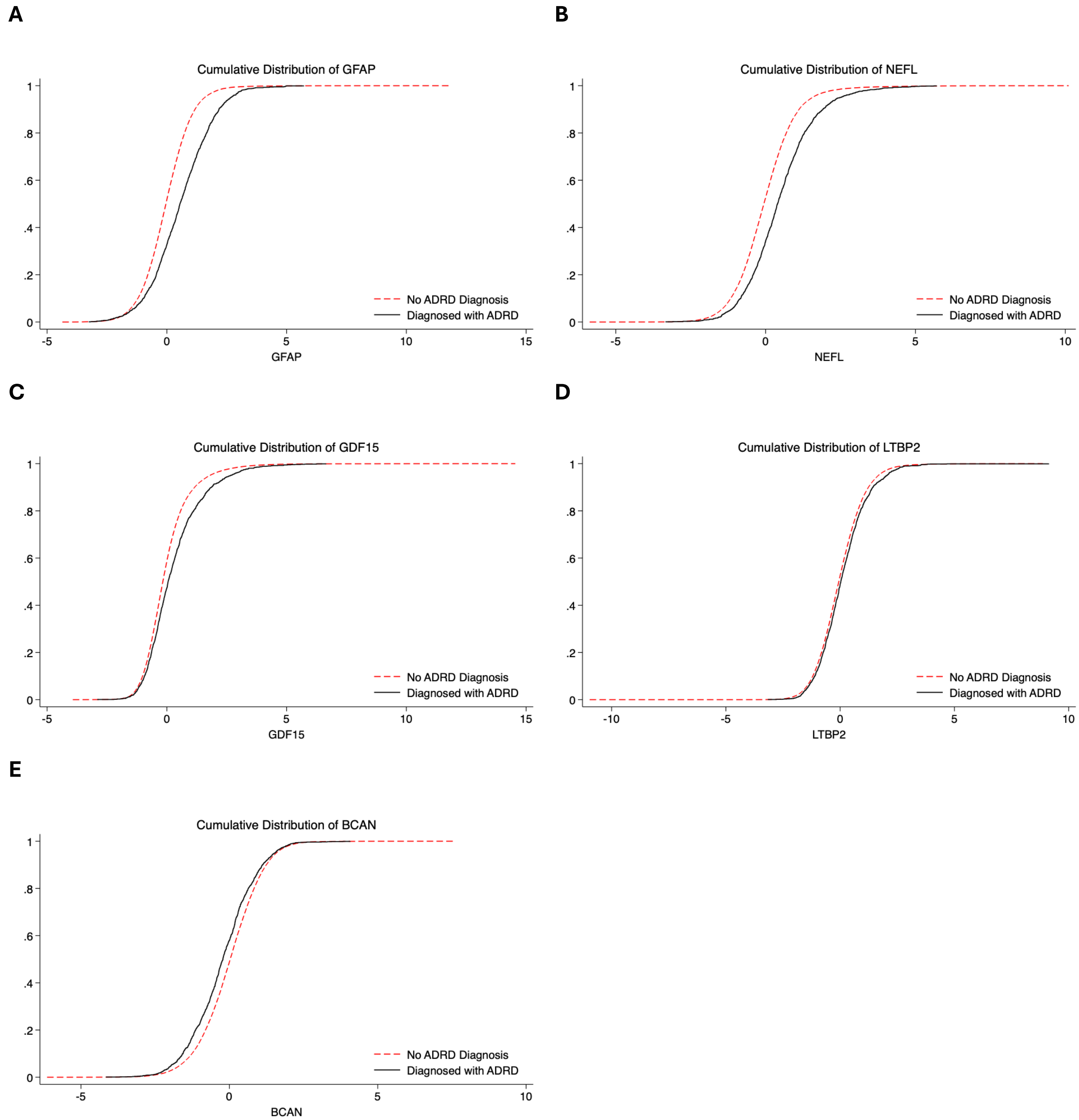

Appendix Figure S2. Dementia Outcome is Associated with Plasma Proteins. The figure shows cumulative distribution functions (CDFs) of five plasma proteins – GFAP (**A**), NEFL (**B**), GDF15 (**C**), LTBP2 (**D**), and BCAN (**E**) – that have been implicated in ADRD (2). Separate CDFs are shown for those who had been diagnosed with dementia by December of 2022 (solid black) and for those who had not been diagnosed (dashed red). The plasma proteins were measured at baseline between 2006 and 2010. Contrary to the other four plasma proteins, BCAN is protective against ADRD. The UK Biobank chose a random sample of its participants and made the proteomic data available for this random sample. That is the sample we use in these analyses. After being adjusted for age, the outcomes were standardized to have a mean of 0 and a standard deviation of 1.

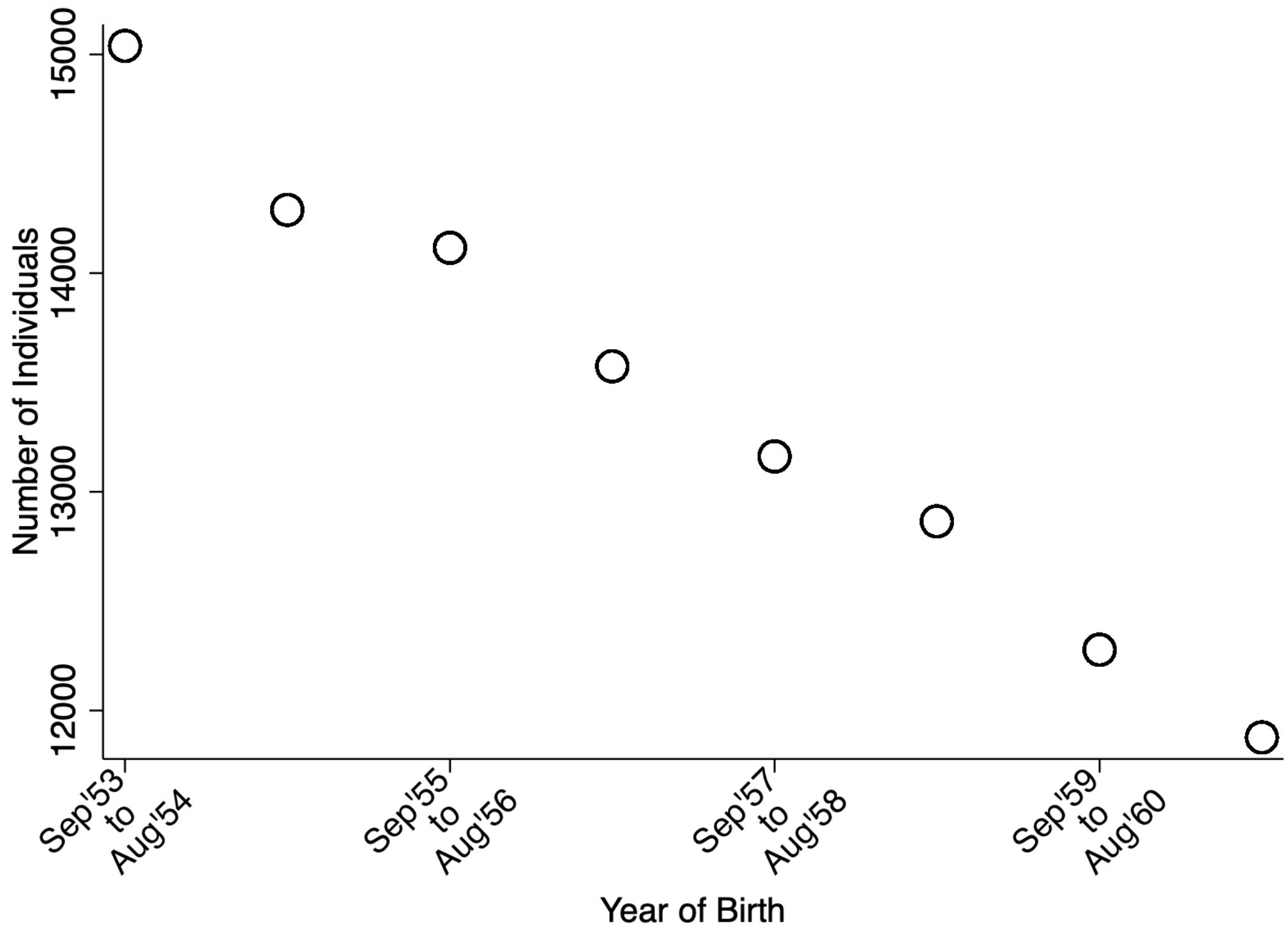

Appendix Figure S3. McCrary Test. The figure shows that we pass the McCrary Test. It shows the number of study participants by year of birth. Year of birth runs from September 1 of a given year to August 31 of the following year. Cohorts born after Year 0 had to stay in school until age 16 while cohorts born before could leave at age 15. The estimated discontinuity of the density is -0.04 with a standard error of 0.024. *N* = 107,200.

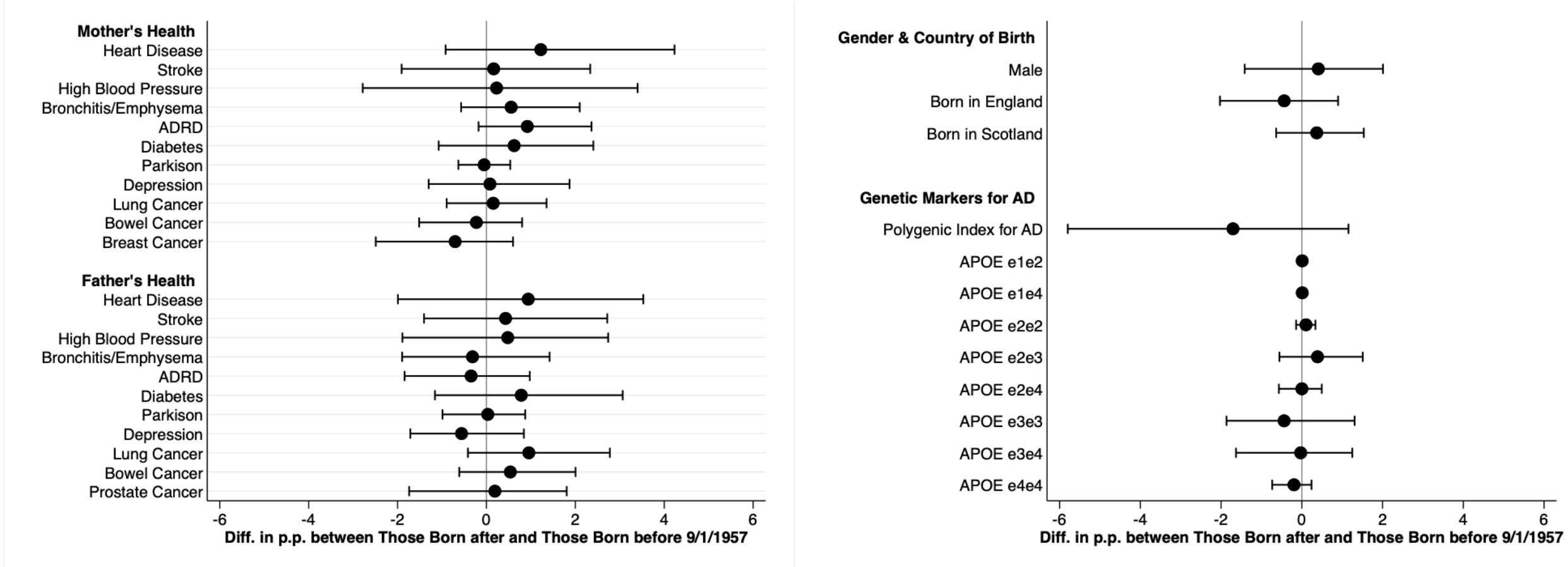

Appendix Figure S4. Balance Test. This figure provides evidence indicating that individuals born right before and right after September 1, 1957 are comparable in terms of pre-reform characteristics. Each row corresponds to a separate regression of the dependent variable listed in the row on an indicator for being born on or after September 1, 1957. All regressions also include exact date of birth to control for birth cohort trends. Separate birth cohort trends are estimated for those born before and those born after September 1, 1957. The markers show point estimates, that is, the coefficient on the indicator for being born on or after September 1, 1957. Estimates are in percentage point units. The brackets represent robust bias-corrected 95% confidence intervals (1, 3), while the point estimates are uncorrected. Because the bias correction is applied only to the confidence intervals, the point estimates (markers) are not necessarily centered within the brackets. Optimal bandwidths are selected using the procedure of Calonico et al. (1). We apply triangular kernel weights, which place greater weight on individuals born closer to the cutoff. The polygenic index for AD, which is the only continuous variable, is normalized to have a standard deviation of one.

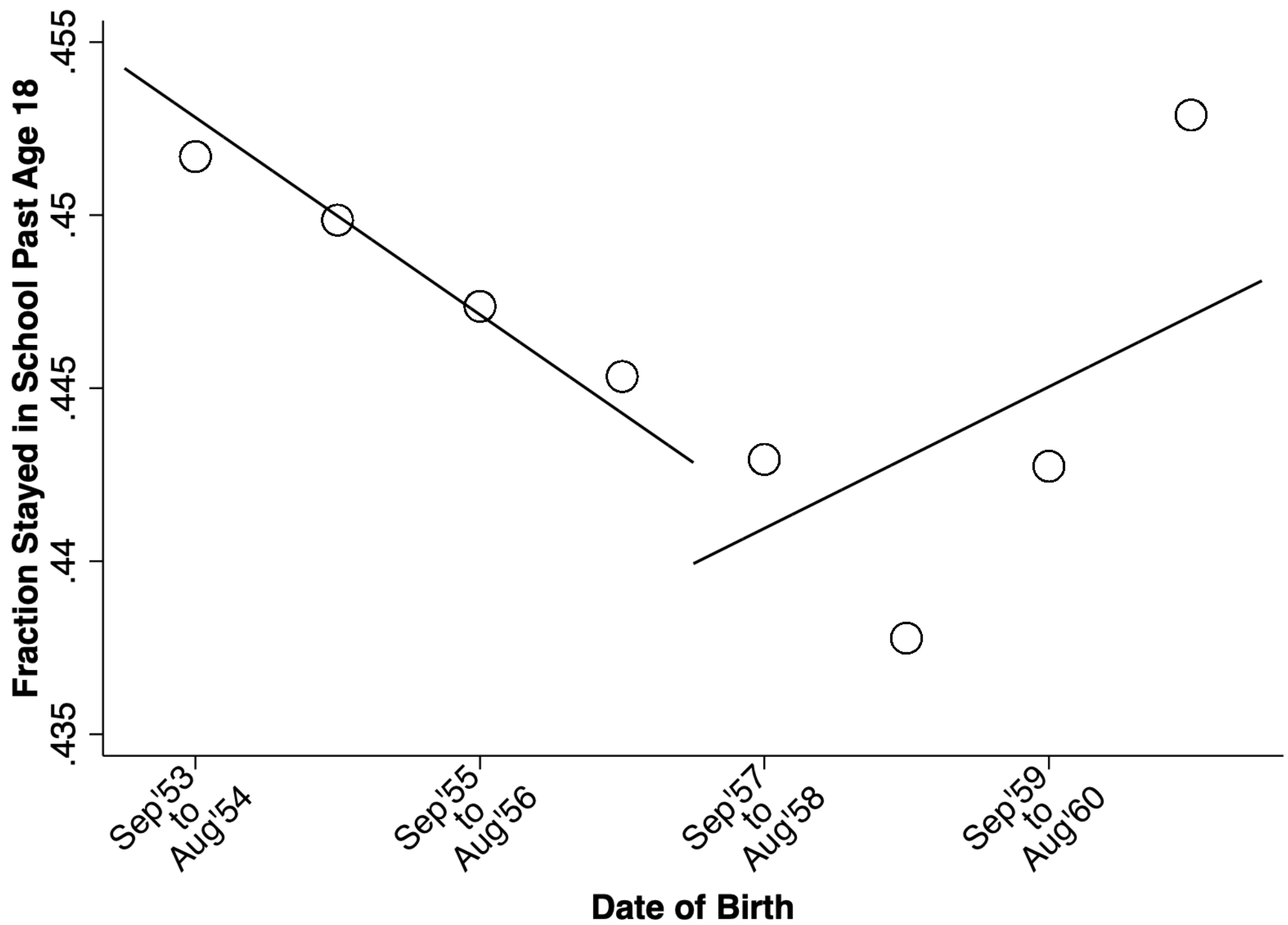

Appendix Figure S5. Reform Had No Effect on Fraction Staying in School Past Age 18. The figure shows that the reform had no effect on the fraction of students staying in school past age 18, which justifies the sample restriction in columns (4) to (6) of Table 1. It shows the fraction of study participants who stayed in school until *at least* age 19 by year of birth. Year of birth runs from September 1 of a given year to August 31 of the following year. Those born in Year 0 were born between September 1, 1957 and August 31, 1958. Cohorts born after Year 0 had to stay in school until age 16 while cohorts born before could leave at age 15. The lines show linear trends in date of birth, which are allowed to be different for those born before and after September 1, 1957. The point estimate is -0.0024 with a standard error of 0.0066 and a p-value of 0.714. The number of observations is 89,259.

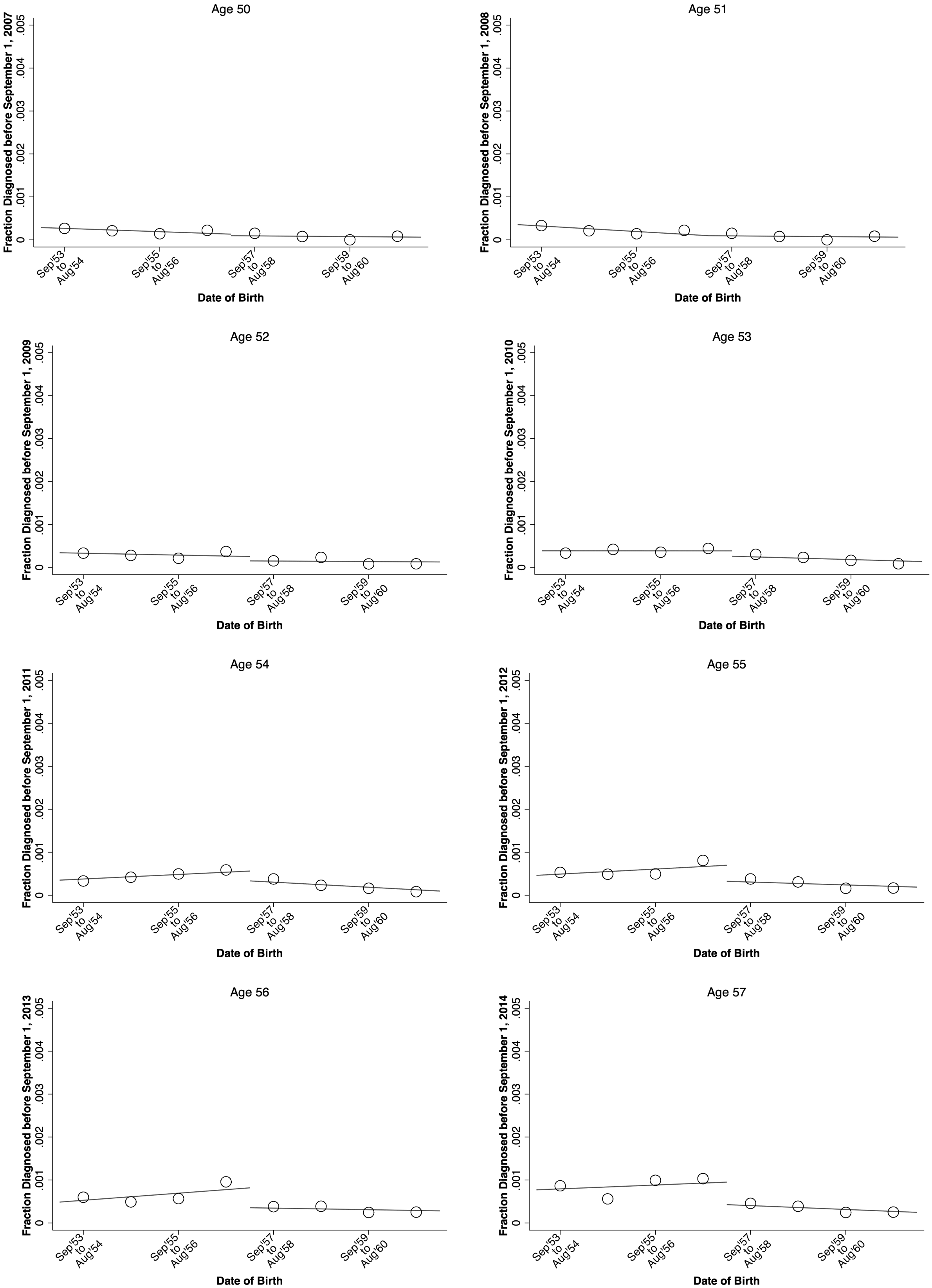
Age 50
Fraction Diagnosed before September 1, 2007
Age 51
Fraction Diagnosed before September 1, 2008
Age 52
Fraction Diagnosed before September 1, 2009
Age 53
Fraction Diagnosed before September 1, 2010
Age 54
Fraction Diagnosed before September 1, 2011
Age 55
Fraction Diagnosed before September 1, 2012
Age 56
Fraction Diagnosed before September 1, 2013
Age 57
Fraction Diagnosed before September 1, 2014
0
.001
.002
.003
.004
.005
Sep'53 to Aug'54
Sep'55 to Aug'56
Sep'57 to Aug'58
Sep'59 to Aug'60
Date of Birth

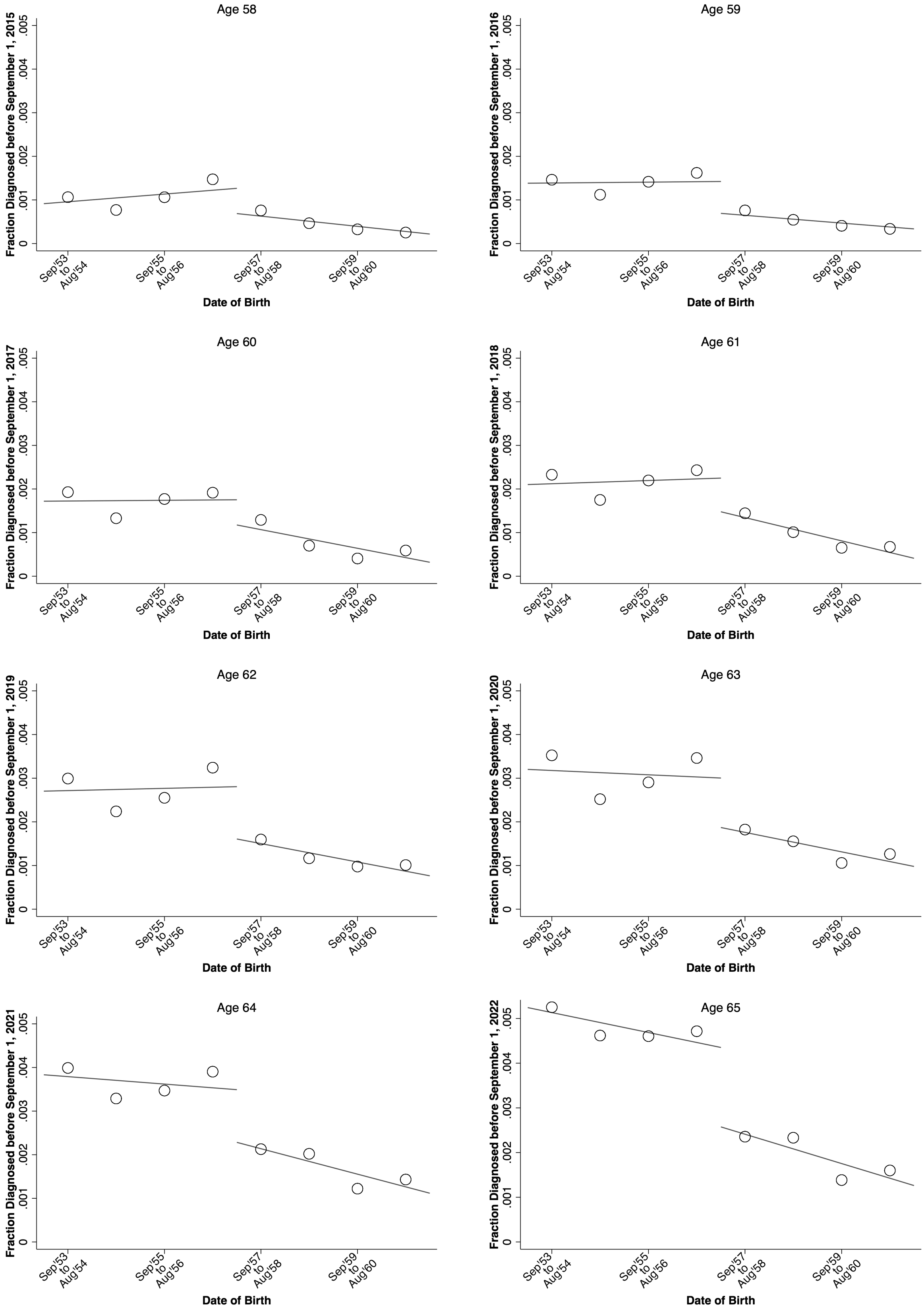

Appendix Figure S6. Effect on Cumulative Incidence at Different Ages. Figure 2A plots the fraction of each birth cohort diagnosed with dementia as of December of 2022. In contrast, the panels above show the fraction diagnosed by September 1 of each year, from 2007 to 2022. The cohort born between September 1, 1957 and August 31, 1958 was the first affected by the reform. The title of each panel indicates the age this cohort had reached by Septmeber 1 of the corresponding year. The lines show linear trends in date of birth, allowed to differ for those born before and after September 1, 1957. The sample is restricted to those born within 4 years of Sep 1, 1957. *N* = 107,200.

Appendix Table S4. Mortality and Selection. This table shows that selective mortality does not drive our results. The 1972 ROSLA may have affected the likelihood of death, which is a potential concern because individuals who live longer are more likely to be diagnosed with ADRD. To investigate this issue, columns (1) and (2) estimate a multinomial logit that distinguishes between three possible outcomes: (a) being alive and having not been diagnosed with ADRD; (b) having died with no ADRD diagnosis; and (c) having been diagnosed with ADRD (the participant may have been alive or not as of December of 2022). Column (1) shows that we still find evidence that those born after September 1, 1957 are less likely to be diagnosed with ADRD. The point estimate in column (2) suggests that those individuals are less likely to have died, but this effect is not statistically significant. Column (3) estimates a regular logit where the omitted group includes both (a) and (b). The comparison of column (3) to column (1) shows that taking selective mortality into account does not affect our main estimate of interest. Columns (1) and (3) show the effects on ADRD risk. Column (2) shows the effect on likelihood of death. "Born after" is an indicator for being born on or after September 1, 1957. All regressions control for date of birth in days and its interaction with the "Born after" indicator variable. Robust standard errors between parentheses. $N = 36{,}530$.

	**Multinomial Logit**		**Logit**
	ADRD	*Dead*	*ADRD*
	(1)	(2)	(3)
Born after	-0.90	-0.04	-0.90
	(0.36)	(0.11)	(0.36)

Appendix Table S5. Linear Probability Model vs Logit – Homogenous Effects of the Reform. This table examines whether the use of a linear probability model (LPM) may contribute to our large estimates, given the low incidence of ADRD in our sample. It re-estimates selected results from the top panel of Table 1 using logistic specifications. In columns (2) and (6), we estimate reduced-form logistic regressions of ADRD on the reform indicator and covariates. In columns (5) and (8), we implement a control function approach: the first stage is estimated using OLS, and the second stage uses the predicted, exogenous change in school-leaving age in a logistic model of ADRD. The odd-numbered columns report the corresponding LPM estimates. The two approaches yield very similar results. All regressions include linear trends in date of birth, allowing for different trends for those born before and after September 1, 1957. In columns (5)–(8), the sample is restricted to individuals who dropped out at age 18 or younger.

	**All**				**Dropped Out at Age 18 or Younger**			
	ADRD (in p.p.)				*ADRD (in p.p.)*			
	Reduced-Form		*Second Stage*		*Reduced-Form*		*Second Stage*	
	LPM	Logit	LPM	Logit	LPM	Logit	LPM	Logit
	(1)	(2)	(3)	(4)	(5)	(6)	(7)	(8)
Born after	-0.20	-0.21			-0.29	-0.26		
	(0.08)	(0.08)			(0.12)	(0.11)		
School-Leaving Age			-1.23	-1.12			-0.88	-1.00
			(0.58)	(0.49)			(0.43)	(0.50)
Mean of Y among Those Born before	0.48	0.48	0.48	0.48	0.60	0.60	0.60	0.60

Appendix Table S6. Linear Probability Model vs Logit – Heterogenous Effect of the Reform on ADRD: The dependent variable is the ADRD cumulative incidence measured in percentage points. "Born after" is an indicator for being born on or after September 1, 1957. All regressions include linear trends in date of birth; those born before and after September 1, 1957 are allowed to have different trends. Odd columns are OLS estimates while even columns present estimates from a logit model. Logit estimates are reported as odds ratios. In columns (3) and (4), the sample is restricted to individuals who dropped out at age 18 or younger. The ADRD genetic risk is standardized to have a standard deviation of one. The last row shows the mean of the dependent variable among those who were born before September 1, 1957. Robust standard errors between parentheses. While common to cluster standard errors on the running variable, we do not need to because our running variable is exact date of birth. Nevertheless, because the running variable is measured very finely, clustering on the running variable produces nearly identical standard errors.

	**All**		**Dropped Out at Age 18 or Younger**	
	ADRD (in p.p.)		*ADRD (in p.p.)*	
	OLS	Logit	OLS	Logit
	(1)	(2)	(3)	(4)
Born after × High Risk	-0.29	-0.74	-0.38	-1.02
	(0.11)	(0.32)	(0.16)	(0.43)
Born after × Low Risk	-0.13	-0.47	-0.14	-0.36
	(0.09)	(0.34)	(0.14)	(0.42)
High Risk	0.21	0.48	0.17	0.36
	(0.11)	(0.25)	(0.17)	(0.31)
P-value Test<br>Effect High Risk = Effect Low Risk	0.247	0.570	0.247	0.272
Bandwidth in Years - High Risk	5.0	5.0	4.9	4.9
Bandwidth in Years - Low Risk	4.1	4.1	4.1	4.1
Observations	112,752	112,752	61,708	61,708
Mean of Y among Those Born before	0.48	0.48	0.61	0.61

Appendix A: The Anderson-Rubin Test and Confidence Intervals

Because two-stage least squares estimates in the just-identified case are effectively ratios of two normally distributed estimates, traditional p-values and confidence intervals have been show to have poor finite sample properties, even when the first stage is well powered (4, 5). For example, the p-values of the reduced form and the two-stage least squares estimates could differ greatly despite corresponding to the same intuitive test. (I.e., under the two-stage least squares assumptions, the instrument should affect the outcome variable if and only if the endogenous variable affects the outcome variable.)

The current guidance is to use an Anderson-Rubin (AR) test (6) as a basis for statistical inference (4). In its simplest form, the AR test is based on the fact that if the structural model for some outcome y_i and some endogenous variable x_i is $y_i = x_i\gamma + e_i$, where e_i is the residual that may be correlated with x_i, then under the assumptions of two-stage least squares, the residual should not be correlated with the instrument z_i. This implies that a possible test of the null hypothesis $\gamma = 0$ is a regression of $e_i = y_i - x_i\gamma = y_i$ onto z_i. Notably, for this null hypothesis, the AR p-value is equivalent to the p-value of the reduced form regression. To identify the AR 95%-confidence interval, we find the set of values of γ_0 such that a regression of $y_i - x_i\gamma_0 = y_i$ onto z_i yields a p-value greater than 0.05, implying that we cannot reject the hypothesis $\gamma = \gamma_0$ under an AR test.

We follow the same principle in our RD application using (1) to select the bandwidth and calculate the confidence intervals for our two-stage least squares estimates. Specifically, for a particular value of γ_0, we calculate the AR test left-hand-side variable $ADRD_i - SLA_i\gamma_0$ and use this as an outcome variable using (1) to estimate the optimal bandwidth and obtain an AR p-value with the same control variables as in our reduced form regression, equation (2) in the main text. We use the p-value corresponding to $\gamma_0 = 0$ for the p-value of the two-stage least squares estimate, and we use a grid search to identify the boundaries of the AR 95%-confidence interval.

Appendix B: Measuring genetic risk of ADRD

As a measure of one's genetic risk factor for developing ADRD, we constructed an ADRD polygenic index (PGI). Our PGI was constructed using two different types of genetic variation. The first type is Single Nucleotide Polymorphisms (SNPs). SNPs are locations in the human genome where individuals differ by a single genetic marker. At most SNPs, people can have one of two possible genetic variants. In genetic data, one of these two possible genetic variants is arbitrarily chosen as the "reference allele". Because a person has two copies of each chromosome, they will either have 0, 1, or 2 copies of the reference allele. The number of reference alleles an individual has at a SNP is called *their genotype for that SNP*.

The second type of variation is the APOE variants. APOE is a gene on chromosome 19 that can be classified into four different variants, numbered APOE1 to APOE4. Each person has two copies of the APOE gene, one for each of their copies of chromosome 19. Individuals with some APOE variants are at much greater risk of developing ADRD—e.g., having two copies of APOE4 is associated with a 10-fold higher risk. We quantify a person's APOE genotype with a set of indicator variables corresponding to each possible pair of APOE variants a person may have (e.g., an indicator for having one copy of APOE3 and one copy of APOE4).

Our PGI is a weighted sum of millions of an individual's genotypes:

$$\sum_j g_{ij} w_j + \sum_k APOE_{ik} \lambda_k$$

where i indexes the individual, j indexes a SNP, and k indexes the different possible pairs of APOE variants a person may have. The variable g_{ij} is individual i's genotype at SNP j while $APOE_{ik}$ is an indicator for whether individual i has the k-th pair of APOE variants. The weights assigned to the different SNPs, w_j, are constructed to maximize the power of the variation across SNPs to predict the outcome of interest – in our case, ADRD. The weights for the SNP genotypes are based on the largest Genome Wide Association Study (GWAS)[1] for Alzheimer's disease that omits the UK Biobank (7).[2] We use a simple model to estimate the weights for the pair of APOE

[1] A GWAS scans the entire genome and estimates associations between individual genotypes and an outcome of interest. Specifically, a GWAS is a series of regressions of some outcome onto the genotype of each SNP, one at a time, and a set of covariates which normally include sex, age, and the first several principal components of the genetic data.

[2] There are several methods for producing PGI weights from GWAS coefficients, but each of them transforms the GWAS coefficients in a way that is meant to account for the correlation structure that exists in the genome. The GWAS associations corresponding to the HapMap3 SNPs were transformed to account for linkage disequilbrium (LD) using the SBayesR method (8), and the EUR subsample of the 1000 Genomes Project data as a reference panel (9). Due to the outsized effect of the APOE region of ADRD risk, we omitted the region on chromosome 19 from basepair position 44.4-46.5Mb (10).

variants, λ_k (see Appendix Table B1 below).[3,4] Because APOE has such a large effect on ADRD incidence, much of the variation in our PGI was driven by APOE (58% of those in the top half had at least one E4 allele, while only 0.02% of those in the bottom half did). Importantly, our PGI is fixed at conception and therefore cannot be affected by the school reform.

Appendix Table B1. ADRD Risk from Genetic Factors. The table shows the association of different genetic measures with ADRD risk. The genetic measures are a polygenic index for AD, indicators for having different APOE genetic markers, and the composite measure that includes both this polygenic component and the APOE genetic markers. This composite measure was constructed based on the results shown in column (3). The AD PGI and the composite measure are standardized to have a standard deviation of one. Columns (1) to (4) show results for study participants born before May 3, 1954 (*N* = 273,712). Columns (5) to (8) show results for those born within 4 years of September 1, 1957 (*N* = 98,778). To construct our composite PGI which also incorporates the effect of the APOE region of the genome, we first follow the approach of Lumsden et al. (12) to call the APOE variant for each person in the full UKB sample. We then regress our measure of ADRD onto the PGI and indicator variables for each person's APOE variant (e.g., ε3ε3, ε3ε4, ε2ε4, etc.) for the individuals in the UKB that are not in the UKB analysis sample. The coefficients from this regression along with the PGI and APOE variant calls are then used to produce the composite PGI in the analysis sample.

[3] To avoid overfitting, these weights were estimated using a set of UK Biobank participants outside our optimal bandwidth.

[4] Following the standard practice, we restrict the sample to individuals whose genetic principal components cluster with the EUR subsample of a genomics reference panel, the 1000 Genomes Project. Due to Euro-centric bias in data collection, most currently published GWASs, including (7), are based on samples with "European" ancestries. As a result, PGIs based on currently available GWAS coefficients are substantially more predictive in European-ancestry samples (11) and may not generalize to groups with African, Asian, Hispanic, or other non-European ancestries. This sample restriction reduces the correlation between a person's genotypes and their environment—such a correlation could produce omitted variable bias in estimates of the effect of genetic variables, such as PGIs, on various outcomes.

	Born Earlier than May 3, 1954				Born within 4 Years of September 1, 1957			
	ADRD (in p.p.)				*ADRD (in p.p.)*			
	(1)	(2)	(3)	(4)	(5)	(6)	(7)	(8)
AD PGI	1.55		0.49		0.10		0.03	
	(0.04)		(0.04)		(0.02)		(0.03)	
Composite Measure				2.16				0.15
				(0.06)				(0.03)
ε1ε2		-1.98	-2.21			-0.29	-0.32	
		(0.04)	(0.04)			(0.02)	(0.03)	
ε1ε4		-1.98	-3.25			-0.29	-0.36	
		(0.04)	(0.20)			(0.02)	(0.07)	
ε2ε2		-0.53	-0.34			0.20	0.22	
		(0.29)	(0.29)			(0.28)	(0.28)	
ε2ε3		-0.35	-0.25			-0.05	-0.04	
		(0.08)	(0.08)			(0.05)	(0.05)	
ε2ε4		1.33	0.83			0.11	0.08	
		(0.22)	(0.22)			(0.13)	(0.13)	
ε3ε4		3.10	2.50			0.14	0.11	
		(0.09)	(0.10)			(0.05)	(0.06)	
ε4ε4		11.41	10.22			0.93	0.86	
		(0.42)	(0.43)			(0.23)	(0.24)	
R^2	0.0080	0.0150	0.0160	0.0160	0.0003	0.0007	0.0007	0.0006

In principle, the relationship between dementia and the PGI, as captured by δ_3 and η_3 in equations (5) and (6), could be confounded by environmental factors. More specifically, imagine we ran the following regression:

$$ADRD_i = \kappa_0 + \kappa_1 G_i + \chi_i. \tag{7}$$

It would yield a biased estimate of the causal effect of the PGI on ADRD if the PGI was correlated for example with the socioeconomic circumstances into which one was born. However, due to the properties of Mendelian inheritance, the genes that one inherits are randomly assigned conditional on the genes of the one's parents (13–15). Therefore, one could obtain an unbiased estimate of this causal effect by estimating the following alternative specification:

$$ADRD_i = \pi_0 + \pi_1 G_i + \mu_i + \varphi_i. \tag{8}$$

where μ_i is a fixed effect specific to individual i and individual i's full siblings. In equation (8), this fixed effect controls for the common parental genotypes for the siblings.

We leveraged this approach in Appendix Table B2 to investigate whether there is empirical support for the hypothesis that the relationship between dementia and the PGI that we have estimated was confounded by environmental factors. In particular, we estimated equation (7) and (8) using a subsample of UKB participants who are siblings. We find that the coefficients on the PGI are similar in the two specifications. We cannot reject the hypothesis that they are the same (p-value 0.402). We interpret this finding as suggestive evidence that the effects of the PGI shown in Figure 3 and in the bottom panel of Table 1 reflect causal genetic effects.

Appendix Table B2. Causal Genetic Effects. This table shows that the associations between dementia and genetic risk shown in the bottom panel of Table 1 and in Figure 4 can be given a causal interpretation. In principle, the association between dementia and genetic risk could be confounded by omitted environmental factors associated with both. However, one can exploit that the genetic variation across (full) siblings is random to isolate causal genetic effects. In order to exploit this variation, this exercise is conducted with UK Biobank participants whose siblings also participated in the study. Column (1) estimates the association between dementia (measured in percentage points) and our measure of one's genetic risk factor for developing ADRD, the AD polygenic index (PGI). Column (2), which includes family fixed effects, estimates the causal effect of the AD PGI (also known as "direct genetic effects") on dementia. The last column of the table formally tests whether the coefficient on the genetic risk in column (2) is statistically different from the coefficient on the genetic risk estimated in column (1). We cannot reject the hypothesis of equality, which leads us to conclude that the associations between dementia and genetic risk shown in the bottom panel of Table 1 and in Figure 3 can be given a causal interpretation. Robust standard errors in column (1). In column (2), standard errors clustered at the family level.

	ADRD (in p.p.)		P-Value Test
	(1)	(2)	(1) = (2)
Genetic Risk	1.13	0.99	0.402
	(0.10)	(0.16)	
Constant	1.86	1.85	-
	(0.07)	(2.2E-3)	
Family Fixed Effects?	No	Yes	
Mean of Dependent Variable	1.84	1.84	
Number of Individuals	38,518	38,518	
Number of Families	-	19,029	

Appendix C: Table 1 Extended Notes

Table 1 Notes: The dependent variables are indicated in the column headings. ADRD cumulative incidence is measured in percentage points. "Born after" is an indicator for being born on or after September 1, 1957. All regressions include linear trends in date of birth; those born before and after September 1, 1957 are allowed to have different trends. Columns (1), (2), (4), and (5) report OLS estimates. Columns (3) and (6) report two-stage least squares estimates, where "Born after" is used as an instrument for school-leaving age. In columns (4) to (6), the sample is restricted to individuals who dropped out at age 18 or younger. In the top panel, optimal bandwidths are selected using the procedure of (1). Robust standard errors are reported in parentheses and robust bias-corrected 95% confidence intervals in brackets (1, 16). We follow the convention in (1), and present point estimates that are not bias-corrected, which explains why they are not centered within the (bias-corrected) confidence intervals. The last row in each panel reports the mean of the dependent variable among individuals born before September 1, 1957. In the bottom panel, "High Risk" and "Low Risk" correspond to individuals in the top and bottom halves of the distribution of the genetic risk measure. In columns (1), (2), (4), and (5) of the bottom panel, the p-value corresponds to a test of whether the coefficients on Born after × High Risk and Born after × Low Risk are equal. In columns (3) and (6) of the bottom panel, the p-value corresponds to a test of whether the coefficients on School-Leaving Age × High Risk and School-Leaving Age × Low Risk are equal. In the bottom panel, optimal bandwidths are selected using the procedure of (16). We apply triangular kernel weights, which place greater weight on individuals born closer to the cutoff. While it is common to cluster standard errors by the running variable, we do not need to do so because the running variable is exact date of birth. Nevertheless, because the running variable is measured very finely, clustering on the running variable produces nearly identical standard errors.

References

1. S. Calonico, M. D. Cattaneo, R. Titiunik, Robust nonparametric confidence intervals for regression-discontinuity designs. *Econometrica* **82**, 2295–2326 (2014).

2. Y. Guo, *et al.*, Plasma proteomic profiles predict future dementia in healthy adults. *Nat. Aging* **4**, 247–260 (2024).

3. S. Calonico, M. D. Cattaneo, R. Titiunik, Optimal data-driven regression discontinuity plots. *J. Am. Stat. Assoc.* **110**, 1753–1769 (2015).

4. M. P. Keane, T. Neal, A practical guide to weak instruments. *Annu. Rev. Econ.* **16**, 185–212 (2024).

5. J. Angrist, M. Kolesár, One instrument to rule them all: The bias and coverage of just-ID IV. *J. Econom.* **240**, 105398 (2024).

6. T. W. Anderson, H. Rubin, Estimation of the parameters of a single equation in a complete system of stochastic equations. *Ann. Math. Stat.* **20**, 46–63 (1949).

7. B. W. Kunkle, *et al.*, Author Correction: Genetic meta-analysis of diagnosed Alzheimer's disease identifies new risk loci and implicates Aβ, tau, immunity and lipid processing. *Nat. Genet.* **51**, 1423–1424 (2019).

8. L. R. Lloyd-Jones, *et al.*, Improved polygenic prediction by Bayesian multiple regression on summary statistics. *Nat. Commun.* **10**, 5086 (2019).

9. L. Clarke, *et al.*, The 1000 Genomes Project: data management and community access. *Nat. Methods* **9**, 459–462 (2012).

10. E. Baker, V. Escott-Price, Polygenic Risk Scores in Alzheimer's Disease: Current Applications and Future Directions. *Front. Digit. Health* **2**, 14 (2020).

11. A. R. Martin, *et al.*, Clinical use of current polygenic risk scores may exacerbate health disparities. *Nat. Genet.* **51**, 584–591 (2019).

12. A. L. Lumsden, A. Mulugeta, A. Zhou, E. Hyppönen, Apolipoprotein E (APOE) genotype-associated disease risks: a phenome-wide, registry-based, case-control study utilising the UK Biobank. *EBioMedicine* **59** (2020).

13. R. S. Spielman, R. E. McGinnis, W. J. Ewens, Transmission test for linkage disequilibrium: the insulin gene region and insulin-dependent diabetes mellitus (IDDM). *Am. J. Hum. Genet.* **52**, 506 (1993).

14. D. B. Allison, Transmission-disequilibrium tests for quantitative traits. *Am. J. Hum. Genet.* **60**, 676 (1997).

15. A. I. Young, *et al.*, Mendelian imputation of parental genotypes improves estimates of direct genetic effects. *Nat. Genet.* **54**, 897–905 (2022).

16. S. Calonico, M. D. Cattaneo, M. H. Farrell, F. Palomba, R. Titiunik, Treatment effect heterogeneity in regression discontinuity designs. arXiv [Preprint] (2025). https://arxiv.org/abs/0806.3533 (Accessed 22 September 2026).

Replication Package

Education Reduces Dementia Incidence and Mitigates Genetic Risk

Silvia H. Barcellos, Leandro S. Carvalho, Kenneth M. Langa, Sneha Nimmagadda, and Patrick Turley

The UK Biobank data used in this study are available to eligible researchers through an application to UK Biobank. Researchers must meet UK Biobank's eligibility requirements and obtain approval for a health-related research project that is in the public interest. Information on eligibility, access requirements, and the application process is available from UK Biobank at https://www.ukbiobank.ac.uk/use-our-data/apply-for-access/.

All code is Stata. Put both do-files and the data in the same working directory, `cd` into it, and run `clean_data.do` first, then `analysis.do`.

1. Data

All individual-level data come from the UK Biobank (UKB) and are **not included** in this package. They are proprietary: researchers must apply to UK Biobank for access (https://www.ukbiobank.ac.uk/enable-your-research). Our analyses were conducted under **UKB Application Number 15666**. Both input files are one row per participant and are keyed on `n_eid`, the UKB participant identifier.

raw_data.dta — UKB phenotypic extract

Variables follow the `n_<field>_<instance>_<array>` (or `ts_` for dates) naming convention. The fields the code reads are:

Field	Variable(s) in code	Used for
33, 34, 52	`ts_33_0_0, n_34_0_0, n_52_0_0`	Date, year, and month of birth (running variable DoB, centered at 1 Sep 1957)
31	`n_31_0_0`	Sex
1647	`n_1647_0_0`	Country of birth
3659	`n_3659_0_0`	Year of immigration (for age at immigration)
845	`n_845_0_0`	Age completed full-time education (school-leaving age, SLA)
6138	`n_6138_0_0–n_6138_0_5`	Qualifications (used only to set SLA = 22 for college)
738	`n_738_0_0`	Household income bracket

Field	Variable(s) in code	Used for
42018	ts_42018_0_0	Date of all-cause dementia report (ADRD outcome)
42000, 42006	ts_42000_0_0, ts_42006_0_0	Dates of myocardial infarction and stroke
42016	ts_42016_0_0	Date of COPD report
20110, 20107	n_20110_0_0–n_20110_0_10, n_20107_0_0–n_20107_0_9	Illnesses of mother and of father (parental ADRD)
93, 94	n_93_0_0, n_93_0_1, n_94_0_0, n_94_0_1	Manual systolic and diastolic blood pressure
4079, 4080	n_4079_0_0, n_4079_0_1, n_4080_0_0, n_4080_0_1	Automated diastolic and systolic blood pressure
21001, 23104	n_21001_0_0, n_23104_0_0	BMI (two measures, averaged)
2443, 4041	n_2443_0_0, n_4041_0_0	Doctor-diagnosed diabetes; gestational diabetes only
30750	n_30750_0_0	HbA1c (mmol/mol), converted to %
1239	n_1239_0_0	Current tobacco smoking

genetic_data.dta — genetic variables

Variable	Description
AD_PGI	SNP-based polygenic index for Alzheimer's disease. Weights from Kunkle et al. (2019), the largest AD GWAS that excludes the UK Biobank. Non-missing only for participants whose genetic principal components cluster with the European-ancestry (EUR) subsample of the 1000 Genomes reference panel.
e1e2, e1e4, e2e2, e2e3, e2e4, e3e4, e4e4	Indicators for the participant's pair of APOE variants. The omitted category is the modal e3e3 pair.

data_for_analysis.dta

Built by `clean_data.do` and read by `analysis.do`.

2. Code

clean_data.do

Reads `raw_data.dta`, merges `genetic_data.dta`, constructs all analysis variables, applies the sample restrictions, and saves `data_for_analysis.dta`.

Sample restrictions. Born in England, Scotland, or Wales, or immigrated at age 14 or younger; non-missing school-leaving age.

Key constructed variables:

- `DoB` — date of birth in days relative to 1 Sep 1957 (running variable); `after` = 1 if `DoB` ≥ 0; `DoBafter` = `DoB` × `after`; `YoB` — year of birth running 1 September to 31 August, 0 for the first treated cohort.
- `SLA` — school-leaving age, set to 22 for participants reporting a college degree; `edu16` = 1 if `SLA` ≥ 16.
- `dementia` — diagnosed with ADRD by December 2022 (field 42018 non-missing).
- `AD_parent` — either parent reported to have developed ADRD (code 10 in fields 20110 / 20107); set to 0 only for respondents who reported at least one other listed illness or “none of the above”, so that non-responders stay missing.
- `optimal_bw` — 1 if `DoB` ∈ [−1217, 1217), the optimal bandwidth reported in the paper.
- `composite_risk` — composite measure of genetic risk for ADRD. Obtained by regressing `dementia` on `AD_PGI` and the APOE pair indicators among participants born before the bandwidth window (`optimal_bw == 0 & DoB < 0`, i.e. born before 3 May 1954), predicting the linear index for everyone, and standardizing it within the bandwidth. Estimating the weights outside the analysis window avoids overfitting.
- `group` — 1 for below-median and 2 for above-median `composite_risk`. The median is computed on the estimation sample (`YoB` between −4 and 3) and then applied to the remaining cohorts.
- Outcomes and ADRD risk factors: `Inc_more_18k / _31k / _52k / _100k` (household income thresholds), `bps` / `bpd` (mean systolic and diastolic blood pressure), `hypertension1` (≥130/80) and `hypertension2` (≥140/90), `BMI` and `obese` (BMI ≥ 30), `diabetes_diagnosis` (excluding gestational-only), `HbA1c` and `diabetes_HbA1c` (≥ 6.5%), `smokes_now`, `stroke`, `infarcation`, `stroke_infarcation`, `COPD`.

analysis.do

Defines two plotting programs and then produces the exhibits in order.

Section	Produces
`homogenous_RD`	Program: RD scatter plus separate pre- and post-cutoff linear fits, for one group
`heterogenous_RD`	Program: the same figure for two groups, plotted together

Section	Produces
Figure 1	A: fraction who stayed in school until age 16. B: fraction whose parents developed ADRD (smoothness check)
Figure 2	A: cumulative ADRD incidence by date of birth. B: the same, by sex
Table 1, top panel	First stage (SLA), reduced form (dementia), and 2SLS, for the full sample and for those who left school at 18 or younger
Table 1, bottom panel	The same three specifications interacted with group, using rdhte for bandwidths and bias-corrected confidence intervals, plus tests of equality across the two genetic-risk groups
Anderson-Rubin	Grid search over candidate 2SLS coefficients to construct AR confidence intervals (see note below)
Figure 3	A: effect on edu16 by genetic-risk group. B: effect on dementia by genetic-risk group
Figure 4	A: intent-to-treat effects on income and ADRD risk factors (coefplot, in percentage points). B: the same, separately by genetic-risk group

All figures restrict to participants born within four years of the cutoff (YoB from −4 to 3). Regressions use the `rdrobust` / `rdhte` optimal bandwidth with triangular weights; the do-file displays results on screen rather than exporting them.

3. Software

Stata 17 or later, plus these user-written commands:

```
net install rdrobust,
from(https://raw.githubusercontent.com/rdpackages/rdrobust/master/stata) replace

net install rdhte,    from(https://raw.githubusercontent.com/rdpackages/rdhte/main/stata)
replace

ssc install coefplot
```

4. Notes on running the code

- **The Anderson-Rubin section is run manually, four times.** The block contains four commented-out parameter sets (full sample and the `SLA < 19` subsample, lower and upper bound each). Uncomment one at a time and rerun the block; each pass grid-searches for the value of the coefficient at which the AR p-value crosses 0.05. Note that the "full sample, upper bound" set is currently left uncommented while its closing `*/` remains, and there is a stray `*/` just above the Table 1 bottom panel; both need to be tidied before the file runs top to bottom.

- `egen BMI = rmean(...)` uses the pre-Stata 8 name for `rowmean` and will need updating on current Stata versions.
- In the `rdhte` calls the second group's bandwidth is read from `e(h)[2,1]`; confirm this matches the layout of `e(h)` in the installed version of `rdhte`.
- The 2SLS estimates in the bottom panel of Table 1 reuse the bandwidth and weights from the preceding `dementia` loop iteration, which is intentional.

STATA do-files:

```
*****************************

*** clean_data.do

*****************************


use n_eid ts_33_0_0 n_738_0_0 n_6138_0_* n_845_0_0 n_3659_0_0 n_1647_0_0 n_52_0_0 n_34_0_0 n_31_0_0 n_2443_0_0 n_4041_0_0 n_93_0_0 n_93_0_1 n_94_0_0 n_94_0_1 n_4079_0_0 n_4079_0_1 n_4080_0_0 n_4080_0_1 n_23104_0_0 n_21001_0_0 n_2443_0_0 n_4041_0_0 n_1239_0_0 n_20110_0_* n_20107_0_* ts_42000_0_0 ts_42006_0_0 ts_42016_0_0 ts_42018_0_0 n_30750_0_0  using "raw_data", clear

merge 1:1 n_eid using "genetic_data", keep(master match) nogen


*****************************

*** Gender

*****************************


ren n_31_0_0 male


*************************

***     Date of birth

*************************


ren n_52_0_0  month_birth

ren n_34_0_0  year_birth

ren ts_33_0_0 date_birth


gen DoB = date_birth - td(01sep1957) // we substract td(01sep1957) such that DoB is 0 at Sep 1, 1957
```

```
*************************
***    Country of birth
*************************

ren n_1647_0_0 country_of_birth

*************************
***  Year of Immigration
*************************

ren n_3659_0_0  year_immigration
        replace year_immigration = .        if year_immigration < 0

gen age_immigration = year_immigration - year_birth

*********************************************
*** Year and Month of Birth
*********************************************

gen MoB = (year_birth*12 + month_birth) - (1957*12 + 9)
gen YoB = floor(MoB/12)
drop MoB

*****************************
*** Post and post-trend
*****************************

gen after=(DoB>=0)
gen DoBafter  = DoB*after
```

```
*****************************
*** School leaving age
*****************************

ren n_845_0_0 SLA

gen college=.
foreach entry of numlist 0/5 {
        replace college=1 if n_6138_0_`entry'==1
}
replace SLA=.    if SLA<0
replace SLA=22   if college==1

gen edu16 = SLA >= 16 if SLA < .

drop college n_6138_0_*

*******************************************
** Average total household income before tax
*******************************************

replace n_738_0_0 = .      if n_738_0_0 < 0
gen Inc_less_18k = 0       if n_738_0_0 < .
        replace Inc_less_18k = 1   if n_738_0_0 == 1
gen Inc_more_31k = 0       if n_738_0_0 < .
        replace Inc_more_31k = 1 if n_738_0_0 >= 3 & n_738_0_0 <= 5
gen Inc_more_52k = 0       if n_738_0_0 < .
        replace Inc_more_52k = 1 if n_738_0_0 >= 4 & n_738_0_0 <= 5
gen Inc_more_100k = 0      if n_738_0_0 < .
```

```
	replace Inc_more_100k = 1		if n_738_0_0 == 5

gen Inc_more_18k = 1 - Inc_less_18k

drop n_738_* Inc_less_18k

*****************************
*** Blood pressure
*****************************

egen bps1 = rowmean(n_4080_0_0 n_93_0_0)
egen bpd1 = rowmean(n_4079_0_0 n_94_0_0)

egen bps2 = rowmean(n_4080_0_1 n_93_0_1)
egen bpd2 = rowmean(n_4079_0_1 n_94_0_1)

egen bps = rowmean(bps1 bps2)
egen bpd = rowmean(bpd1 bpd2)

**************************
* Hypertension
**************************

gen hypertension1 = (bps >= 130 | bpd >= 80)		if bps < . & bpd < .
gen hypertension2 = (bps >= 140 | bpd >= 90)		if bps < . & bpd < .

*****************************
*** Anthropometrics
*****************************
```

```
egen BMI = rmean(n_23104_0_0 n_21001_0_0)

gen obese=(BMI>=30) if BMI<.

*****************************
*** Diabetes
*****************************

gen diabetes_diagnosis = .
	replace diabetes_diagnosis = 0	if n_2443_0_0 == 0
	replace diabetes_diagnosis = 1	if n_2443_0_0 == 1

	replace diabetes_diagnosis = 0	if diabetes_diagnosis == 1 & n_4041_0_0 == 1


	*****************************
	*** HbA1c
	*****************************

	gen HbA1c = (0.09148*n_30750_0_0) + 2.152
	gen diabetes_HbA1c		=	(HbA1c >= 6.5) if HbA1c < .

*****************************
*** Smoking
*****************************

gen smokes_now = .
	replace smokes_now = 0	if n_1239_0_0 == 0
	replace smokes_now = 1	if n_1239_0_0 == 1 | n_1239_0_0 == 2
```

```
*****************************
*** Dementia
*****************************

gen dementia=0
        replace dementia=1 if ts_42018_0_0 < .

*************************************
*                 Stroke & Infarcation          *
*************************************

gen infarcation = (ts_42000_0_0 < .)

gen stroke = (ts_42006_0_0 < .)

gen stroke_infarcation = (stroke == 1 | infarcation == 1) if stroke < . & infarcation < .

*************************************
*                          COPD                *
*************************************

gen COPD = (ts_42016_0_0 < .)

***********************************************************
*  IDENTIFYING INDIVIDUALS WHOSE PARENTS DEVELOPED DEMENTIA  *
***********************************************************

gen mother_AD = .
forvalues i = 0/10 {
```

```
	replace mother_AD = 0	if	n_20110_0_`i' == 1  | ///
					n_20110_0_`i' == 2  | ///
					n_20110_0_`i' == 8  | ///
					n_20110_0_`i' == 6  | ///
					n_20110_0_`i' == 9  | ///
					n_20110_0_`i' == - 17

}

forvalues i = 0/10 {

	replace mother_AD = 1	if n_20110_0_`i' == 10

}

gen father_AD = .
forvalues i = 0/9 {

	replace father_AD = 0	if	n_20107_0_`i' == 1  | ///
					n_20107_0_`i' == 2  | ///
					n_20107_0_`i' == 8  | ///
					n_20107_0_`i' == 6  | ///
					n_20107_0_`i' == 9  | ///
					n_20107_0_`i' == - 17

}

forvalues i = 0/9 {
```

```
		replace father_AD = 1		if  n_20107_0_`i' == 10

	}


	gen AD_parent = .
		replace AD_parent = 0		if father_AD == 0 & mother_AD == 0
		replace AD_parent = 1		if father_AD == 1 | mother_AD == 1


	drop father_AD mother_AD

		*************************************
		*			Sample Restriction			*
		*************************************

		keep if (country_of_birth == 1 | country_of_birth == 2 | country_of_birth == 3 | age_immigration
<= 14)

		* Born in England, Scotland, or Wales or immigrated at age 14 or younger

		drop age_immigration

		*************************************
		* Drop if school leaving age missing  *
		*************************************

		drop if SLA >= .

	*************************************
	*			Optimal Bandwidth		*
```

```
*************************************

gen optimal_bw = (DoB >= -1217 & DoB < 1217)

*********************************************************
* using PGI only for those from European genetic ancestry  *
*********************************************************

summ AD_PGI
replace AD_PGI = (AD_PGI - r(mean))/r(sd)

***************************************************
*          COMPOSITE MEASURE OF GENETIC RISK FOR DEMENTIA   *
***************************************************

reg dementia AD_PGI e1e2 e1e4 e2e2 e2e3 e2e4 e3e4 e4e4 if optimal_bw == 0 & DoB < 0

predict composite_risk, xb

summ composite_risk if optimal_bw == 1
replace composite_risk = (composite_risk - r(mean))/r(sd)

***************************************************
*           BELOW/ABOVE MEDIAN GENETIC RISK FOR DEMENTIA    *
***************************************************

cap drop group
xtile group = composite_risk  if YoB >= -4 & YoB <= 3 , nquantiles(2)

_pctile composite_risk if YoB >= -4 & YoB <= 3 , nquantiles(2)
```

```
local cutoff = r(r1)

replace group = 1 if composite_risk < `cutoff' & (YoB < -4 | YoB > 3)
replace group = 2 if composite_risk > `cutoff' & (YoB < -4 | YoB > 3) & composite_risk < .

****************************************************
****************************************************
save "data_for_analysis", replace



************************************************************
*** analysis.do
****************************
////////////////////////////////////////
//                                                          //
//      PROGRAM THAT CREATES RD FIGURES   //
//                  FOR A SINGLE GROUP          //
//                                                          //
////////////////////////////////////////

capture program drop homogenous_RD
program define    homogenous_RD

    preserve

        *1: dependent variable
        *2: title of y-axis

        qui {
```

```
keep if YoB >= -4 & YoB <= 3

cap drop x
cap drop min
cap drop max

bysort YoB: egen min = min(DoB)
bysort YoB: egen max = max(DoB)
gen x = (min + max)/2
replace x = round(x)

reg `1' DoB         if DoB <  0
global pre_a = _b[_cons]
global pre_b = _b[DoB]
reg `1' DoB if DoB >= 0
global post_a = _b[_cons]
global post_b = _b[DoB]

bysort YoB: egen depvar = mean(`1')
collapse (mean) `1' (mean) depvar (mean) YoB (mean) x, by(date_birth)

gen DoB = date_birth - td(01sep1957)



gen pre  = $pre_a  + ($pre_b  * DoB) if DoB <= 0
gen post = $post_a + ($post_b * DoB) if DoB >= 0


}
```

```
        twoway  (scatter depvar    x, msymbol(Oh)    mcolor(black) msize(vlarge) mlwidth(vthin))   ///
                (line    pre DoB, lcolor(black))  ///
                (line   post DoB, lcolor(black))  ///
                , ytitle("{bf:`2'}") legend(off) ///
                xtitle("{bf:Date of Birth}") ///
                graphregion(fcolor(white)) plotregion(lwidth(none)) ///
                scheme(s1mono) ///
                ylabel(, nogrid) ///
                title("", size(medium)) ///
                xlabel(-1280 "Sep'53 " -1180 "to      "  -1080 "Aug'54    " ///
                  -550  "Sep'55 " -450  "to      "   -350 "Aug'56    " ///
                   190  "Sep'57 "  290  "to      "    390 "Aug'58    " ///
                   910  "Sep'59 "  1010 "to      "   1110 "Aug'60    " ///
                        , angle(45) noticks) ///
                xtick(-1279 -548 182 913)

    restore

end

////////////////////////////////////////
//                                                  //
//      PROGRAM THAT CREATES RD FIGURES   //
//                  FOR TWO GROUPS              //
//                                                  //
////////////////////////////////////////

capture program drop heterogenous_RD
program define    heterogenous_RD
```

```
preserve

	*1: dependent variable
	*2: title of y-axis
	*3: variable identifying different groups
	*4: legend 1
	*5: legend 2
	*6: color1
	*7: color2


	qui {

		keep if YoB >= -4 & YoB <= 3

		cap drop x
		cap drop min
		cap drop max

		bysort YoB: egen min = min(DoB)
		bysort YoB: egen max = max(DoB)
		gen x = (min + max)/2
		replace x = round(x)


		reg `1' DoB		if DoB <  0 & `3' == 1
		global group1_pre_a = _b[_cons]
		global group1_pre_b = _b[DoB]
		reg `1' DoB if DoB >= 0 & `3' == 1
		global group1_post_a = _b[_cons]
```

```
global group1_post_b = _b[DoB]


reg `1' DoB          if DoB <  0 & `3' == 2
global group2_pre_a = _b[_cons]
global group2_pre_b = _b[DoB]
reg `1' DoB if DoB >= 0 & `3' == 2
global group2_post_a = _b[_cons]
global group2_post_b = _b[DoB]

bysort YoB `3': egen depvar = mean(`1')
collapse (mean) `1' (mean) depvar (mean) YoB (mean) x, by(date_birth `3')

gen DoB = date_birth - td(01sep1957)


gen group1_pre  = $group1_pre_a  + ($group1_pre_b  * DoB) if DoB <= 0 & `3'
== 1
gen group1_post = $group1_post_a + ($group1_post_b * DoB) if DoB >= 0 &
`3' == 1
gen group2_pre  = $group2_pre_a  + ($group2_pre_b  * DoB) if DoB <= 0 & `3'
== 2
gen group2_post = $group2_post_a + ($group2_post_b * DoB) if DoB >= 0 &
`3' == 2

}

twoway  (scatter depvar    x if `3' == 1, msymbol(X)   mcolor(`6')  msize(vlarge)
)            ///
        (scatter depvar    x if `3' == 2, msymbol(Oh) mcolor(`7')
msize(vlarge) mlwidth(vthin)) ///
        (line    group1_pre DoB if DoB <= 0 & `3' == 1, lcolor(`6'))
///
```

```
                                        (line   group1_post DoB if DoB >= 0 & `3' == 1, lcolor(`6'))
///
                                        (line    group2_pre DoB if DoB <= 0 & `3' == 2, lcolor(`7'))
///
                                        (line   group2_post DoB if DoB >= 0 & `3' == 2, lcolor(`7'))
///
                                        , ytitle("{bf:`2'}") ///
                                        xtitle("{bf:Date of Birth}") ///
                                        graphregion(fcolor(white)) plotregion(lwidth(none)) ///
                                        scheme(s1mono) ///
                                        ylabel(, nogrid) ///
                                        title("", size(medium)) ///
                                        legend(order(1 "`4'" 2 "`5'") row(1) region(lstyle(none))) ///
                                        xlabel(-1280 "Sep'53 " -1180 "to      "  -1080 "Aug'54    " ///
                                           -550  "Sep'55 " -450  "to      "   -350 "Aug'56    " ///
                                            190  "Sep'57 "  290  "to      "    390 "Aug'58    " ///
                                            910  "Sep'59 "  1010 "to      "   1110 "Aug'60    " ///
                                                , angle(45) noticks) ///
                                        xtick(-1279 -548 182 913) //

            restore

      end

      ////////////////////////////////////////
      //                                                              //
      //                    FIGURE 1                    //
      //                                                              //
      ////////////////////////////////////////

      use "data_for_analysis", clear
```

```
homogenous_RD edu16 "Fraction Stayed in School until Age 16"

homogenous_RD AD_parent "Fraction Whose Parents Developed ADRD"

////////////////////////////////////////
//                                                              //
//                  FIGURE 2A                    //
//                                                              //
////////////////////////////////////////

homogenous_RD dementia "Cumulative ADRD Incidence in December 2022"

////////////////////////////////////////
//                                                              //
//                  FIGURE 2B                    //
//                                                              //
////////////////////////////////////////

gen gender = .
	replace gender = male + 1

heterogenous_RD dementia "Cumulative ADRD Incidence in Dec 2022" gender "Female" "Male" "pink"
"blue"

////////////////////////////////////////
//                                                              //
//            TABLE 1 TOP PANEL                  //
//                                                              //
////////////////////////////////////////
```

```
use "data_for_analysis", clear

cap drop  __000000

replace edu16 = edu16 * 100
replace dementia = dementia * 100


foreach depvar in SLA dementia {

        rdrobust `depvar' DoB, p(1)

        local h = e(h_l)
        local lb = e(ci_l_rb)
        local ub = e(ci_r_rb)

        mean `depvar' if DoB > -`h' & DoB < 0

        local mean = _b[`depvar']


        cap drop weight
        gen weight = .
                replace weight = 1  - (abs(DoB)/`h')
                replace weight = 0 if weight < 0

        reg `depvar' after DoB DoBafter if DoB > -`h' & DoB < `h' [aw = weight], r

}
```

```
rdrobust dementia DoB, fuzzy(SLA) p(1)

local h = e(h_l)

cap drop weight
gen weight = .
        replace weight = 1  - (abs(DoB)/`h')
        replace weight = 0 if weight < 0


ivregress 2sls dementia DoB DoBafter (SLA = after) if DoB > -`h' & DoB < `h' [aw = weight], r



foreach depvar in SLA dementia {

        rdrobust `depvar' DoB if SLA < 19, p(1)

        local h = e(h_l)
        local lb = e(ci_l_rb)
        local ub = e(ci_r_rb)

        mean `depvar' if DoB > -`h' & DoB < 0 & SLA < 19

        local mean = _b[`depvar']

        cap drop weight
        gen weight = .
                replace weight = 1  - (abs(DoB)/`h')            if SLA < 19
```

```
			replace weight = 0						if weight < 0 & 
SLA < 19

		reg `depvar' after DoB DoBafter if DoB > -`h' & DoB < `h' & SLA < 19 [aw = weight], r

	}

	rdrobust dementia DoB if SLA < 19, fuzzy(SLA) p(1)

	local h = e(h_l)

	cap drop weight
	gen weight = .
		replace weight = 1  - (abs(DoB)/`h')if SLA < 19
		replace weight = 0						if weight < 0 & SLA < 19


	ivregress 2sls dementia DoB DoBafter (SLA = after) if DoB > -`h' & DoB < `h' & SLA < 19 [aw = weight], 
r



	*/
	///////////////////////////////////////
	//									//
	//			TABLE 1 BOTTOM PANEL			//
	//									//
	///////////////////////////////////////

	gen top = group == 2 if group < .
	gen low = group == 1 if group < .
```

```
gen top_after = top * after
gen low_after = low * after


gen top_SLA = SLA * top
gen low_SLA = SLA * low

gen DoB_top = DoB * top
gen DoB_low = DoB * low

gen DoBafter_top = DoBafter * top
gen DoBafter_low = DoBafter * low



foreach depvar in SLA dementia {


	rdhte `depvar' DoB, covs_hte(i.group) p(1)

	local h1 = e(h)[1,1]
	local h2 = e(h)[2,1]

	local lb1 = e(tau_ci_lb)[1,1]
	local ub1 = e(tau_ci_ub)[1,1]
	local lb2 = e(tau_ci_lb)[2,1]
	local ub2 = e(tau_ci_ub)[2,1]
```

```
		rdhte_lincom 2.group - 1.group

		local other_test =  e(rdhte_lincom_pv)



		mean `depvar' if DoB < 0 & ((DoB > -`h1' & low == 1)|(DoB > -`h2' & top == 1))


		local mean = _b[`depvar']


		cap drop weight

			gen weight = .

				replace weight = 1  - (abs(DoB)/`h1')		if low == 1

				replace weight = 1  - (abs(DoB)/`h2')		if top == 1

				replace weight = 0 if weight < 0



		reg `depvar' top_after low_after top DoB_low DoBafter_low DoB_top DoBafter_top if ((DoB > -`h1' & DoB < `h1' & low==1) | (DoB > -`h2' & DoB < `h2' & top==1)) [aw=weight], r


		test (top_after = low_after)

		local pvalue = r(p)


	}



	ivregress 2sls dementia top DoB_low DoBafter_low DoB_top DoBafter_top (top_SLA low_SLA = top_after low_after)  if ((DoB > -`h1' & DoB < `h1' & low==1) | (DoB > -`h2' & DoB < `h2' & top==1)) [aw=weight], r


	test (top_SLA = low_SLA)

	local pvalue = r(p)
```

```
foreach depvar in SLA dementia {

        rdhte `depvar' DoB if SLA < 19, covs_hte(i.group) p(1)

        local h1 = e(h)[1,1]
        local h2 = e(h)[2,1]

        local lb1 = e(tau_ci_lb)[1,1]
        local ub1 = e(tau_ci_ub)[1,1]
        local lb2 = e(tau_ci_lb)[2,1]
        local ub2 = e(tau_ci_ub)[2,1]

        rdhte_lincom 2.group - 1.group
        local other_test =  e(rdhte_lincom_pv)

        mean `depvar' if DoB < 0 & ((DoB > -`h1' & low == 1)|(DoB > -`h2' & top == 1)) & SLA < 19

        local mean = _b[`depvar']

        cap drop weight
                gen weight = .
                        replace weight = 1  - (abs(DoB)/`h1')      if low == 1       & SLA < 19
                        replace weight = 1  - (abs(DoB)/`h2')      if top == 1       & SLA < 19
                        replace weight = 0                                           if weight
< 0     & SLA < 19

        reg `depvar' top_after low_after top DoB_low DoBafter_low DoB_top DoBafter_top if ((DoB > -
`h1' & DoB < `h1' & low==1) | (DoB > -`h2' & DoB < `h2' & top==1)) & SLA < 19 [aw=weight], r
```

```
test (top_after = low_after)

local pvalue = r(p)

}

ivregress 2sls dementia top DoB_low DoBafter_low DoB_top DoBafter_top (top_SLA low_SLA = top_after low_after)  if ((DoB > -`h1' & DoB < `h1' & low==1) | (DoB > -`h2' & DoB < `h2' & top==1)) & SLA < 19 [aw=weight], r

test (top_SLA = low_SLA)

local pvalue = r(p)

////////////////////////////////////////
//                                                                      //
//                    ANDERSON-RUBIN (AR)          //
//                    CONFIDENCE INTERVAL                    //
//                                                                      //
////////////////////////////////////////

use dementia DoB SLA using "data_for_analysis", clear

cap drop  __000000

replace dementia = dementia * 100

////////////////////////////////////////
//                                                                      //
//                    FULL SAMPLE                              //
```

```
//                              LOWER BOUND                                    //
//                                                                             //
////////////////////////////////////////

/*
local beta_min  = -2.72
local beta_max  = -2.7
local beta_step =  -0.001
*/

////////////////////////////////////////
//                                                                             //
//                              FULL SAMPLE                          //
//                              UPPER BOUND                          //
//                                                                             //
////////////////////////////////////////


local beta_min  = -.19
local beta_max  = -.17
local beta_step =  -0.001
*/

////////////////////////////////////////
//                                                                             //
//  THOSE WHO DROPPED OUT bef AGE 19  //
//                              LOWER BOUND                                    //
//                                                                             //
////////////////////////////////////////
```

```
/*
keep if SLA < 19

local beta_min  = -1.77
local beta_max  =  -1.76
local beta_step =  -0.001
*/

////////////////////////////////////////
//                                                                                              //
//  THOSE WHO DROPPED OUT bef AGE 19  //
//                              UPPER BOUND                              //
//                                                                                              //
////////////////////////////////////////
/*
keep if SLA < 19

local beta_min  = -.16
local beta_max  =  -.15
local beta_step =  -0.001
*/




* Count number of grid points
local ngrid = floor((`beta_max' - `beta_min') / `beta_step') + 1

* Create temporary file to store results
tempfile results
postfile handle beta0 pval using `results', replace
```

```
forvalues b = `beta_max'(`beta_step')`beta_min' {

	cap drop _u_resid
	gen _u_resid = dementia - (SLA * `b')

	* Run rdrobust on residualized outcome
	capture rdrobust _u_resid DoB, p(1)

	if _rc == 0 {

		local pval  = e(pv_rb)

		post handle (`b') (`pval')

		di "beta0 = " %6.3f `b' "  p-value = " %6.4f `pval'
	}
	else {
		di "beta0 = " %6.3f `b' "  rdrobust FAILED"
		post handle (`b') (.)
	}
}

postclose handle


use `results', clear

///////////////////////////////////////
```

```
//                                                  //
//                    FIGURE 3A                //
//                                                  //
////////////////////////////////////////

use "data_for_analysis", clear

heterogenous_RD edu16 "Fraction Stayed in School until Age 16" group "Low Genetic Risk for ADRD" "High Genetic Risk for ADRD" "black" "red"



////////////////////////////////////////
//                                                  //
//                    FIGURE 3B                //
//                                                  //
////////////////////////////////////////

heterogenous_RD dementia "Fraction Diagnosed with ADRD" group "Low Genetic Risk for ADRD" "High Genetic Risk for ADRD" "black" "red"



////////////////////////////////////////
//                                                  //
//                    FIGURE 4A                //
//                                                  //
////////////////////////////////////////

use "data_for_analysis", clear

cap drop __000000
```

```
local i = 1
foreach depvar in   Inc_more_18k ///
                                        Inc_more_31k ///
                                        Inc_more_52k ///
                                        Inc_more_100k ///
                                        diabetes_HbA1c ///
                                        diabetes_diagnosis ///
                                        obese ///
                                        hypertension1  ///
                                        hypertension2  ///
                                        stroke_infarcation  ///
                                        smokes_now  ///
                                        COPD ///
                                         {

     replace `depvar' = `depvar' * 100


     rdrobust `depvar' DoB, p(1)

     matrix coef_`i'  =  e(tau_cl)
     matrix lb_`i'    =  e(ci_l_rb)
     matrix ub_`i'    =  e(ci_r_rb)
     matrix colnames coef_`i' = `depvar'
     matrix colnames lb_`i'   = `depvar'
     matrix colnames ub_`i'   = `depvar'

     local h = e(h_l)
```

```
		cap drop weight
		gen weight = .
			replace weight = 1  - (abs(DoB)/`h')
			replace weight = 0 if weight < 0

		reg `depvar' after DoB DoBafter if DoB > -`h' & DoB < `h' [aw=weight], r

		local i = `i' + 1

	}

	coefplot (matrix(coef_1)		, ci((lb_1  ub_1))  mcolor(black) msymbol(O) msize(medlarge)
ciopts(recast(rcap) color(black))	offset(0)) ///
			(matrix(coef_2)		, ci((lb_2  ub_2))  mcolor(black) msymbol(O) msize(medlarge)
ciopts(recast(rcap) color(black))	offset(0)) ///
			(matrix(coef_3)		, ci((lb_3  ub_3))  mcolor(black) msymbol(O) msize(medlarge)
ciopts(recast(rcap) color(black))	offset(0)) ///
			(matrix(coef_4)		, ci((lb_4  ub_4))  mcolor(black) msymbol(O) msize(medlarge)
ciopts(recast(rcap) color(black))	offset(0)) ///
			(matrix(coef_5)		, ci((lb_5  ub_5))  mcolor(black) msymbol(O) msize(medlarge)
ciopts(recast(rcap) color(black))	offset(0)) ///
			(matrix(coef_6)		, ci((lb_6  ub_6))  mcolor(black) msymbol(O) msize(medlarge)
ciopts(recast(rcap) color(black))	offset(0)) ///
			(matrix(coef_7)		, ci((lb_7  ub_7))  mcolor(black) msymbol(O) msize(medlarge)
ciopts(recast(rcap) color(black))	offset(0)) ///
			(matrix(coef_8)		, ci((lb_8  ub_8))  mcolor(black) msymbol(O) msize(medlarge)
ciopts(recast(rcap) color(black))	offset(0)) ///
			(matrix(coef_9)		, ci((lb_9  ub_9))  mcolor(black) msymbol(O) msize(medlarge)
ciopts(recast(rcap) color(black))	offset(0)) ///
			(matrix(coef_10)	, ci((lb_10 ub_10))	mcolor(black) msymbol(O)
msize(medlarge) ciopts(recast(rcap) color(black))	offset(0)) ///
			(matrix(coef_11)	, ci((lb_11 ub_11))	mcolor(black) msymbol(O)
msize(medlarge) ciopts(recast(rcap) color(black))	offset(0)) ///
			(matrix(coef_12)	, ci((lb_12 ub_12))	mcolor(black) msymbol(O)
msize(medlarge) ciopts(recast(rcap) color(black))	offset(0)) ///
			,	headings(Inc_more_18k = "{bf: Household Income}" ///
```

```
diabetes_HbA1c = "{bf: Risk Factors}") ///
coeflabels(Inc_more_18k = "Income ≥ £18,000" ///
Inc_more_31k = "Income ≥ £31,000" ///
Inc_more_52k = "Income ≥ £52,000" ///
Inc_more_100k = "Income ≥ £100,000" ///
diabetes_HbA1c = "Diabetes (HbA1c Test)" ///
diabetes_diagnosis = "Diabetes (Ever Diagnosed)" ///
obese = "Obesity" ///
hypertension1 = "Stage 1 Hypertension"  ///
hypertension2 = "Stage 2 Hypertension"  ///
stroke_infarcation = "Stroke or Myocardial Infarction"  ///
smokes_now = "Current Smoker"  ///
COPD = "COPD" ///
, labsize(small)) ///
xline(0, lcolor(gs8) lwidth(thin)) scheme(s2mono) ///
graphregion(fcolor(white)) ylabel(, noticks) ///
xtitle("{bf:Effect of Raising of The Minimum School-Leaving Age (in p.p.)}", size(small)) ///
xlabel(-4(2)4, labsize(small)) xscale(range(-4 4)) ///
grid(none) ///
legend(off) //


////////////////////////////////////////
//                                                        //
//                 FIGURE 4B               //
//                                                        //
////////////////////////////////////////

use "data_for_analysis", clear
```

```
cap drop __000000

gen top = group == 2 if group < .
gen low = group == 1 if group < .

gen top_after = top * after
gen low_after = low * after

gen top_SLA = SLA * top
gen low_SLA = SLA * low

gen DoB_top = DoB * top
gen DoB_low = DoB * low

gen DoBafter_top = DoBafter * top
gen DoBafter_low = DoBafter * low

local i = 1
foreach depvar in   diabetes_HbA1c ///
                                        diabetes_diagnosis ///
                                        obese ///
                                        hypertension1  ///
                                        hypertension2  ///
                                        stroke_infarcation  ///
                                        smokes_now  ///
```

```
COPD ///
{

replace `depvar' = `depvar' * 100



rdhte `depvar' DoB, covs_hte(i.group) p(1)


local h_low = e(h)[1,1]
local h_top = e(h)[2,1]


matrix coef_low_`i'  =  e(tau_hat)[1,1]
matrix coef_top_`i'  =  e(tau_hat)[1,2]
matrix lb_low_`i' = e(tau_ci_lb)[1,1]
matrix ub_low_`i' = e(tau_ci_ub)[1,1]
matrix lb_top_`i' = e(tau_ci_lb)[2,1]
matrix ub_top_`i' = e(tau_ci_ub)[2,1]


matrix colnames coef_low_`i' = `depvar'
matrix colnames lb_low_`i'   = `depvar'
matrix colnames ub_low_`i'   = `depvar'
matrix colnames coef_top_`i' = `depvar'
matrix colnames lb_top_`i'   = `depvar'
matrix colnames ub_top_`i'   = `depvar'



local h = e(h_l)


cap drop weight
gen weight = .
```

```
replace weight = 1  - (abs(DoB)/`h_low')      if (DoB > -`h_low' & DoB < `h_low' & group == 1)

replace weight = 1  - (abs(DoB)/`h_top')      if (DoB > -`h_top' & DoB < `h_top' & group == 2)

replace weight = 0 if weight < 0


reg `depvar' top_after low_after top DoB_top DoBafter_top DoB_low DoBafter_low if (DoB > -`h_low' & DoB < `h_low' & group == 1) | (DoB > -`h_top' & DoB < `h_top' & group == 2) [aw=weight], r


local i = `i' + 1


}


local minus_offset = -.125

local plus_offset = .125


coefplot (matrix(coef_low_1)          , ci((lb_low_1  ub_low_1))        mcolor(black) msymbol(O) msize(medlarge) ciopts(recast(rcap) color(black))        offset(`minus_offset')) ///

         (matrix(coef_top_1)          , ci((lb_top_1  ub_top_1))  mcolor(red) msymbol(T) msize(medlarge) ciopts(recast(rcap) color(red))        offset(`plus_offset')) ///

         (matrix(coef_low_2)          , ci((lb_low_2  ub_low_2))        mcolor(black) msymbol(O) msize(medlarge) ciopts(recast(rcap) color(black))        offset(`minus_offset')) ///

         (matrix(coef_top_2)          , ci((lb_top_2  ub_top_2))  mcolor(red) msymbol(T) msize(medlarge) ciopts(recast(rcap) color(red))        offset(`plus_offset')) ///

         (matrix(coef_low_3)          , ci((lb_low_3  ub_low_3))        mcolor(black) msymbol(O) msize(medlarge) ciopts(recast(rcap) color(black))        offset(`minus_offset')) ///

         (matrix(coef_top_3)          , ci((lb_top_3  ub_top_3))  mcolor(red) msymbol(T) msize(medlarge) ciopts(recast(rcap) color(red))        offset(`plus_offset')) ///

         (matrix(coef_low_4)          , ci((lb_low_4  ub_low_4))        mcolor(black) msymbol(O) msize(medlarge) ciopts(recast(rcap) color(black))        offset(`minus_offset')) ///

         (matrix(coef_top_4)          , ci((lb_top_4  ub_top_4))  mcolor(red) msymbol(T) msize(medlarge) ciopts(recast(rcap) color(red))        offset(`plus_offset')) ///

         (matrix(coef_low_5)          , ci((lb_low_5  ub_low_5))        mcolor(black) msymbol(O) msize(medlarge) ciopts(recast(rcap) color(black))        offset(`minus_offset')) ///
```

```
                        (matrix(coef_top_5)                     , ci((lb_top_5  ub_top_5))  mcolor(red)
     msymbol(T) msize(medlarge) ciopts(recast(rcap) color(red))                offset(`plus_offset')) ///

                        (matrix(coef_low_6)                     , ci((lb_low_6  ub_low_6))             mcolor(black)
     msymbol(O) msize(medlarge) ciopts(recast(rcap) color(black))              offset(`minus_offset')) ///

                        (matrix(coef_top_6)                     , ci((lb_top_6  ub_top_6))  mcolor(red)
     msymbol(T) msize(medlarge) ciopts(recast(rcap) color(red))                offset(`plus_offset')) ///

                        (matrix(coef_low_7)                     , ci((lb_low_7  ub_low_7))             mcolor(black)
     msymbol(O) msize(medlarge) ciopts(recast(rcap) color(black))              offset(`minus_offset')) ///

                        (matrix(coef_top_7)                     , ci((lb_top_7  ub_top_7))  mcolor(red)
     msymbol(T) msize(medlarge) ciopts(recast(rcap) color(red))                offset(`plus_offset')) ///

                        (matrix(coef_low_8)                     , ci((lb_low_8  ub_low_8))             mcolor(black)
     msymbol(O) msize(medlarge) ciopts(recast(rcap) color(black))              offset(`minus_offset')) ///

                        (matrix(coef_top_8)                     , ci((lb_top_8  ub_top_8))  mcolor(red)
     msymbol(T) msize(medlarge) ciopts(recast(rcap) color(red))                offset(`plus_offset')) ///

                        ,           headings(diabetes_HbA1c = "{bf: Risk Factors}") ///

                                    coeflabels(Inc_more_18k = "Income ≥ £18,000" ///

                                    Inc_more_31 = "Income ≥ £31,000" ///

                                    Inc_more_52k = "Income ≥ £52,000" ///

                                    Inc_more_100k = "Income ≥ £100,000" ///

                                    diabetes_HbA1c = "Diabetes (HbA1c Test)" ///

                                    diabetes_diagnosis = "Diabetes (Ever Diagnosed)" ///

                                    obese = "Obesity" ///

                                    hypertension1 = "Stage 1 Hypertension"  ///

                                    hypertension2 = "Stage 2 Hypertension"  ///

                                    stroke_infarcation = "Stroke or Myocardial Infarction"  ///

                                    smokes_now = "Current Smoker"  ///

                                    COPD = "COPD" ///

                                    , labsize(small)) ///

                                    xline(0, lcolor(gs8) lwidth(thin)) scheme(s2mono) ///

                                    graphregion(fcolor(white)) ylabel(, noticks) ///

                                    xtitle("{bf:Effect of Raising of The Minimum School-Leaving Age (in p.p.)}",
size(small)) ///

                                    xlabel(-4(2)4, labsize(small)) xscale(range(-4 4)) ///

                                    grid(none) ///
```

```
                legend(order(2 "Low Genetic Risk for ADRD" 4 "High Genetic Risk for
ADRD") row(1) size(small) region(lstyle(none))) //
```
